# Sequential Application of Time-Stratified Demographic, Vital, Clinical–Laboratory, and Microbiology Variables for Accurate and Rapid Identification of Sepsis

**DOI:** 10.3390/diseases14090319

**Published:** 2026-09-01

**Authors:** Krupa Arun Navalkar, José Garnacho-Montero, María Luisa Cantón-Bulnes, José Luís García-Garmendia, Ángel Estella, Adela Fernández-Galilea, Isidro Blanco, Maria Antonia Estecha-Foncea, Marina Gordillo-Resina, Jorge Rodríguez-Gómez, Juan Jesús Pineda-Capitán, Carmen Martínez-Fernández, Ana Escoresca-Ortega, Rosario Amaya-Villar, Juan Mora-Ordóñez, Sara González-Soto, Antonio Gutierrez-Pizarraya, Robert Balk, Russell R. Miller, John P. Burke, Gourang Patel, Jorge P. Parada, Marcus J. Schultz, Brendon P. Scicluna, Emily Blodget, Santhi Kumar, Dayle Sampson, Thomas D. Yager, Roy F. Davis, Silvia Cermelli, Richard B. Brandon

**Affiliations:** 1 Immunexpress Inc., Seattle, WA 98109, USA; roy.d@immunexpress.com (R.F.D.); silvia.c@immunexpress.com (S.C.); richard.b@immunexpress.com (R.B.B.); 2 Intensive Care Unit, Hospital Universitario Virgen del Rocío, 41013 Sevilla, Spain; jgarnachom@gmail.com (J.G.-M.); adelafer_90@hotmail.com (A.F.-G.); nanaesco99@gmail.com (A.E.-O.); ramayavillar@gmail.com (R.A.-V.); 3 Intensive Care Unit, Hospital Universitario Virgen Macarena, 41009 Sevilla, Spain; luisabulnes@hotmail.com; 4 Intensive Care Unit, Hospital San Juan de Dios del Aljarafe, 41930 Sevilla, Spain; joseluis.garciagarmendia@sjd.es (J.L.G.-G.); carmen.martinezfernandez@sjd.es (C.M.-F.); 5 Intensive Care Unit, Hospital Universitario de Jerez de la Frontera, 11407 Cádiz, Spain; angel.estella@gm.uca.es (Á.E.); isidro.blanco.sspa@juntadeandalucia.es (I.B.); 6 Instituto de Investigación e Innovación Biomédica de Cádiz (INiBICA), Department of Medicine and Surgery, University of Cádiz, 11003 Cádiz, Spain; 7 Intensive Care Unit, Hospital Universitario Virgen de la Victoria (Málaga), 29010 Málaga, Spain; mariaa.estecha.sspa@juntadeandalucia.es (M.A.E.-F.); marina.gordillo.sspa@juntadeandalucia.es (M.G.-R.); 8 Intensive Care Unit, Hospital Universitario Reina Sofía (Córdoba), 14004 Córdoba, Spain; jorge.rodriguez.gomez.sspa@juntadeandalucia.es (J.R.-G.); juanjesus.pineda.sspa@juntaandalucia.es (J.J.P.-C.); 9 Intensive Care Unit, Hospital Universitario Regional de Málaga (Málaga), 29010 Málaga, Spain; jumouci@gmail.com (J.M.-O.); saragsoto24@gmail.com (S.G.-S.); 10 Agencia Andaluza de Evaluación de Tecnología Sanitaria, 41092 Sevilla, Spain; boticariors@gmail.com; 11 Rush Medical College and Rush University Medical Center, Chicago, IL 60612, USA; robert_balk@rush.edu; 12 FirstHealth of the Carolinas, Pinehurst, NC 28374, USA; rmiller@firsthealth.org; 13 Intermountain Medical Center, Murray, UT 84107, USA; john.burke@imail.org; 14 School of Medicine, University of Utah, Salt Lake City, UT 84132, USA; 15 Department of Pharmacy Services, University of Chicago Medicine, Chicago, IL 60637, USA; gourang.patel@uchicagomedicine.org; 16 Loyola University Medical Center, Maywood, IL 60153, USA; jparada@lumc.edu; 17 Division of Cardiothoracic and Vascular Anaesthesia & Critical Care Medicine, Department of Anaesthesia, General Intensive Care and Pain Management, Medical University Wien, 1090 Vienna, Austria; marcus.j.schultz@gmail.com; 18 Department of Anaesthesiology, Rescue and Pain Medicine, Cantonal Hospital St. Gallen, HOCH Health Ostschweiz, 9007 St. Gallen, Switzerland; 19 Nuffield Department of Medicine, University of Oxford, Oxford OX3 7BN, UK; 20 Mahidol–Oxford Tropical Medicine Research Unit (MORU), Mahidol University, Bangkok 10400, Thailand; 21 Centre for Molecular Medicine and Biobanking, University of Malta, MSD 2080 Msida, Malta; brendon.scicluna@um.edu.mt; 22 Department of Applied Biomedical Science, Faculty of Health Sciences, Mater Dei Hospital, University of Malta, MSD 2080 Msida, Malta; 23 Keck Hospital of University of Southern California (USC), Los Angeles, CA 90033, USA; eblodge1@hs.uci.edu (E.B.); santhi.kumar@med.usc.edu (S.K.); 24 Los Angeles General Medical Center, Los Angeles, CA 90033, USA; 25 Elysium Health, 434 Broadway, New York, NY 10013, USA; dayle@elysiumhealth.com

**Keywords:** sepsis, systemic inflammatory response syndrome (SIRS), diagnostic accuracy, logistic regression, host response assay, procalcitonin, intensive care, early diagnosis, temporal analysis, SeptiCyte RAPID

## Abstract

Background: Accurate early identification of sepsis remains a major clinical challenge due to its heterogeneous presentation and overlap of clinical signs with the non-infectious systemic inflammatory response syndrome (SIRS). Timely differentiation is crucial for improving patient outcomes, meeting sepsis bundle requirements and reducing inappropriate antimicrobial use. We hypothesized that clinical–laboratory data available within the first three hours of patient presentation could be used to identify patients with sepsis at a clinically useful level of diagnostic accuracy, in lieu of traditional microbiology results which would not become available until at least 12–24 h. Data from two independent studies were used to quantify the diagnostic value of demographic, vital, clinical–laboratory, and microbiological data available at three time points for distinguishing retrospectively diagnosed critically ill patients with either sepsis or non-infectious SIRS. A particular focus of this work was an assessment of the utility of SeptiCyte RAPID (Immunexpress Inc., Seattle, WA, USA) as an aid to sepsis diagnosis, producing actionable data within one hour. Methods: Data from two independent study cohorts were analyzed. The “510(k) cohort” consisted of 419 adult patients in intensive care (ICU) (MARS, VENUS, and NEPTUNE studies). The “Andalusian cohort” consisted of 353 ICU patients from the PANGEA study. Logistic regression models, selected by a greedy search algorithm and validated by repeated cross-validation, were used to determine the contributions of different variables to diagnostic accuracy. Diagnostic performance was quantified by the area under the receiver operating characteristic curve (AUC). Results: For the 510(k) cohort, a baseline AUC of 0.69–0.73 was observed using five to seven vital and demographic variables assessed immediately upon presentation (time T1). The addition of clinical–laboratory variables, in particular SeptiCyte RAPID, within one to three hours post-presentation (time T2) increased the AUC to 0.85–0.86. Finally, the addition of microbiological data 12–24 h post-presentation (time T3) further improved the AUC to 0.90–0.91. Similar results were obtained for the Andalusian cohort. AUC values at the three time points were as follows: At time T1, AUC = 0.67 based solely on vital signs and demographics; at time T2, AUC = 0.87 based on vitals + demographics + SeptiCyte RAPID ± other clinical–laboratory data; at time T3, AUC = 0.93 based on vitals + demographics + SeptiCyte RAPID ± other clinical–laboratory data + microbiology results. For both cohorts, the most significant variables included temperature, mean arterial pressure, respiratory rate, suspected infection site, SeptiCyte RAPID, procalcitonin, confirmed bacterial infection and positive blood culture confirmation. In summary, the AUC for diagnosing sepsis rose progressively from T1 (510(k) 0.69–0.73; Andalusian 0.67) to T2 (510(k) 0.85–0.86; Andalusian 0.87) with the addition of SeptiCyte RAPID to T3 (510(k) 0.90–0.91; Andalusian 0.93) as more clinical information became available over time. Conclusions: The accuracy of identification of sepsis increases markedly as demographics and vital signs are supplemented with clinical–laboratory information, and ultimately with microbiological culture results. The AUC improves in the shortest time within the first three hours when laboratory data, and particularly SeptiCyte RAPID results, become available. Integrating rapid host response testing with SeptiCyte RAPID into time-based diagnostic frameworks may enhance early sepsis recognition, improve antimicrobial stewardship, and support guideline-driven clinical decisions.

## 1. Introduction

Sepsis, a dysregulated host response to infection leading to life-threatening organ dysfunction, remains a major diagnostic challenge due to its heterogeneous presentation and overlap of clinical signs with non-infectious inflammatory conditions including the systemic inflammatory response syndrome (SIRS) [1,2]. 

Diagnostic uncertainty in cases of suspected sepsis may lead to delays in targeted treatment or even to inappropriate treatments. Consequences may include increased hospital readmission rates, long-term cognitive and physical impairments, and increased mortality [3]. Diagnostic uncertainty with respect to sepsis has multiple root causes. Clinical signs of sepsis are often nonspecific, varying with infection site, pathogen, and patient comorbidities. Furthermore, up to one-third of confirmed sepsis cases lack an identifiable infectious source [1]. 

Misdiagnosis at admission is common (~12%), with misdiagnosed patients having more than double the odds of death compared to correctly diagnosed patients (adjusted odds ratio ≈ 2.7–3.0) [4]. These authors concluded that early accurate diagnosis, rather than solely rapid treatment, should be a critical focus in sepsis care, as misdiagnosis contributes to worse outcomes despite adherence to time-sensitive treatment bundles. Improving sepsis diagnostic accuracy therefore has both patient safety implications and significant medicolegal relevance [5,6]. Additionally, misdiagnosis, over-diagnosis, and under-diagnosis contributes substantially to healthcare costs across Organization for Economic Cooperation and Development (OECD) nations, with diagnostic errors estimated to cause serious harm in nearly 10% of U.S. cases annually [7,8].

Despite advances in sepsis management guidelines, early and accurate identification of sepsis remains a major challenge, with standard clinical scoring systems providing only moderate discriminatory power and traditional microbiological results unavailable for one to three days. This creates a window of diagnostic uncertainty during which treatment decisions must be made without adequate precision, with implications for patient safety and antimicrobial stewardship. We hypothesized that clinical and laboratory variables available within the first one to three hours, including SeptiCyte RAPID, could identify patients with sepsis at a clinically useful level of diagnostic accuracy in lieu of microbiology results. To test this hypothesis, we analyzed data from two independent ICU cohorts using a time-stratified framework modeling the sequential accumulation of diagnostic information at three clinically meaningful time points (T1: presentation; T2: 1–3 h; T3: one to three days), aiming to identify the key variables contributing most to accurate and timely sepsis identification. The primary aim of this study was to quantify the incremental diagnostic value of clinical information as it becomes available over time, rather than to evaluate the performance of any individual biomarker in isolation.

## 2. Materials and Methods

### 2.1. Study Cohorts/Datasets

Datasets from two independent study cohorts were used to assess the utility of the conceptual framework (sequential data utilization at times T1, T2, T3). Although the two datasets could not be combined (due to incomplete overlap of variables), the separate analyses were largely concordant and supportive of each other. Factors that prevented directly pooling the two datasets were as follows. Pooling would have provided very few continuous numeric variables in common, specifically SeptiScore, procalcitonin (PCT), lactate, and age, aside from 18 categorical variables in common between both. Several vitals were collected as maximum and minimum values within the first 24 h of ICU admission in the 510(k), which are not the same as a single value collected after ICU admission in the Andalusian cohort. The sepsis framework used for adjudication was sepsis-2 for the 510(k) cohort and sepsis-3 for the Andalusian cohort. The retrospective physician diagnosis (RPD) methodology used for sepsis adjudication was different between the two cohorts: blinded review by three panelists for the 510(k) vs. site investigator/Steering Committee review in the Andalusian cohort. Supplementary Table S3 shows the overlap of variables between the 510(k) and Andalusian cohorts.

510(k) cohort (Dataset #1): This cohort included 419 critically ill adult patients, suspected of sepsis within the first 24 h of ICU admission and displaying at least two SIRS criteria. Final adjudicated diagnoses were sepsis (*n* = 176) or SIRS (*n* = 243), as described below. The cohort comprised a retrospective sub-cohort (*n* = 356) and a prospective sub-cohort (*n* = 63), as previously reported by Balk et al. (2024) [9]. The retrospective sub-cohort was drawn from the observational MARS, VENUS, and VENUS Supplement trials (NCT01905033 and NCT02127502; clinicaltrials.gov (accessed on 17 February 2024)). Recruitment dates were January 2011–December 2013 (MARS, Europe), May 2014–April 2015 (VENUS, USA) and March–August 2016 (VENUS Supplement, USA). The prospective sub-cohort consisted of 63 critically ill adult subjects enrolled in an observational trial (NEPTUNE, NCT05469048; clinicaltrials.gov (accessed on 17 February 2024)) between 26 May 2020 and 25 April 2021 at Emory University/Grady Memorial Hospital (Atlanta, GA, USA), Rush University Medical Center (Chicago, IL, USA) and University of Southern California (USC) Medical Center (with two separate sites, Keck Hospital of USC, and Los Angeles General Medical Center, in Los Angeles, CA, USA). 

Ethics approval for the MARS trial was given by the Medical Ethics Committee of the Amsterdam Medical Center (approval # 10-056C). Ethics approvals for the VENUS trial were given by the relevant Institutional Review Boards as follows: Intermountain Medical Center/Latter Day Saints Hospital (approval # 1024931); Johns Hopkins Hospital (approval # IRB00087839); Rush University Medical Center (approval # 15111104-IRB01); Loyola University Medical Center (approval # 208291); Northwell Healthcare (approval #16-02-42-03). Ethics approvals for the NEPTUNE trial were given by the relevant Institutional Review Boards as follows: Emory University (approval # IRB00115400); Grady Memorial Hospital (approval # 00-115400); Rush University Medical Center (approval # 19101603-IRB01); University of Southern California Medical Center (approval # HS-19-0884-CR001). All subjects, or their legally authorized representatives, gave informed consent for participation in the respective study. All methods used in this study were carried out in accordance with the relevant guidelines and regulations.

Andalusian cohort (Dataset #2): This cohort comprised 353 critically ill ICU patients with final adjudicated diagnoses of sepsis (*n* = 267) or SIRS (*n* = 86), as described below. It was derived from the multicenter prospective PANGEA study, details of which have been published [10]. The study was conducted between 3 March and 20 December 2022, across seven ICUs in Andalusia, Spain, and was coordinated by the Virgen Macarena University Hospital in Seville. Two of the hospitals (Hospital Virgen del Rocío (Seville), Hospital Universitario Regional de Málaga (Málaga)) initiated their studies late (in September and October 2022, respectively) due to organizational problems and delays in approval by the Research Ethics Committees. The study was not registered in any public clinical trial database, as it was an observational diagnostic accuracy study with no therapeutic intervention. The study followed the Standards for Reporting of Diagnostic Accuracy (STARD) guidelines for reporting studies of diagnostic accuracy. All patients aged 18 years or older, admitted to the ICU and with a clinical suspicion of sepsis according to the Sepsis-3 definition were included (*n* = 353). The sepsis vs. SIRS distinction was a retrospective classification determined afterward, and not part of the enrollment criteria. Subjects were excluded if they were pregnant, or if the clinical picture suggestive of sepsis had started more than 48 h previously. Blood samples were collected in PAXgene Blood RNA tubes (PreAnalytiX GmbH, Hombrechtikon, Switzerland), from which 0.9 mL aliquots were pipetted into SeptiCyte RAPID cartridges and run on the Idylla system on-site. 

The study was approved by the Research Ethics Committees (CEI) of the Virgen Macarena–Virgen Rocio University Hospitals on 20 December 2021 (Internal Code, 2662-N-21). The research ethics committees allowed blood sample extractions prior to obtaining written permission, if needed, to enable SeptiCyte RAPID results to be generated as quickly as possible. Written consent from the patient or next of kin was obtained within 48 h of ICU admission, and the blood sample was discarded if written consent was not obtained.

### 2.2. Reference Diagnoses (Comparators)

510(k) cohort: The diagnostic performance of individual and combined variables was evaluated by comparison to a ‘gold standard’ of retrospective sepsis/SIRS clinical adjudication. The adjudication process, termed retrospective physician diagnosis (RPD), was conducted under the sepsis-2 and sepsis-3 definitions and has been previously described [11]. RPD used a panel of three independent expert physicians who did not attend to the patients. Each physician was provided with the same information, including selected vitals, laboratory results, and microbiology, and was asked to provide a diagnostic call of either sepsis or SIRS (forced diagnosis). A positive culture alone was not sufficient for a sepsis call. The RPD panel was blinded to SeptiCyte RAPID and procalcitonin (PCT) results to prevent the panelists’ diagnoses from being influenced by these two variables. This blinding was considered particularly important given the complexity of the host response in sepsis, including thromboinflammatory mechanisms that can confound clinical discrimination of sepsis from non-infectious SIRS [12]. For other variables, such as some vital signs and laboratory variables, an incorporation bias effect is acknowledged and discussed in detail below. These variables were part of the standard clinical data collected in patient charts that the RPD panelists reviewed to make their diagnosis. Inter-observer agreement for the RPD process has been estimated previously using free-marginal kappa (κfree) in a 249-patient subset of the 510(k) cohort and ranged from κfree = 0.50 (respiratory focus) to 0.70–0.74 (non-respiratory, presumptive SIRS) [13]. No equivalent statistics are available for the Andalusian cohort’s site investigator/Steering Committee adjudication.

Andalusian cohort: Patients were categorized as “sepsis” or “sterile inflammation” by two investigators at each participating site who were aware of the clinical, analytical, and microbiological data but blinded to the SeptiCyte results. A Steering Committee of four clinicians reviewed all cases and contacted local investigators in case of doubt. All patients were followed up until death or hospital discharge. 

For both cohorts, an incorporation bias effect is acknowledged for some of the variables and is quantified for both cohorts in Section 3.8. Adjudicators in both cohorts had access to microbiological results, so the T3 AUCs should be read as upper bounds on unbiased diagnostic performance rather than as unbiased estimates. This applies more strongly to the Andalusian cohort, where the microbiology variables alone reach AUC 0.81 (Supplementary Table S14) and no equivalent of the 510(k) rule that a positive culture alone was insufficient for a sepsis call was applied.

### 2.3. Variables Used in the Analyses

Variables were grouped into three categories based on their typical availability after patient presentation. Time T1, available immediately upon presentation: patient history and vital signs. Time T2, available within 1–3 h: clinical–laboratory variables. Time T3, available within 1–3 days: microbiological culture results. Viral PCR/antigen tests (to detect active infections) were run for some but not all patients, based on individual assessment by the care team. The timing of when these tests were ordered and run was not recorded and might likely fall beyond the 1–3 h T2 window. Because of this timing uncertainty, viral PCR/antigen test results, when available, were included in the later T3 time point. 

510(k) cohort: From an initial assessment of 47 different clinical–laboratory variables, 42 of the variables had less than 33.4% missing values and were included in further analyses (see Supplementary Table S1). A missing value cutoff of ~33.4% was a pragmatic balance between limiting the extent of imputation [14] and incorporating as many variables as possible. Missing values for 27 of these 42 variables were imputed as the means of the available values, using the function PCA from the FactoMineR R package version 2.12 by Josse and Husson (2008) [15]. Simple mean imputation was used in preference to multiple imputation as a pragmatic simplification; we note that single imputation understates variance relative to multiple imputation [14]. Missingness in the 510(k) cohort was largely missing-by-design: several variables were not collected by protocol in the VENUS and VENUS Supplement studies, so missingness is with a missing-at-random mechanism. A complete case sensitivity analysis for both cohorts is reported in Supplementary Table S11. The study composition of the 510(k) complete case subset is given in Supplementary Table S11b.

Maximum and minimum values for the clinical–laboratory variables MAP, temperature, HR, WBC, and glucose were recorded over the first 24 h in ICU. These max and min values were used as proxies for data that would ordinarily, in clinical practice, be collected within the first few minutes of presentation (at time T1). In the 510(k) data, maximum and minimum values within 24 h of ICU admission were used as the best available proxy for single values given the constraints of retrospective data collection, consistent with the convention used for APACHE II and SOFA scoring. This is noted as a limitation in Section 4.4. Reassuringly, the Andalusian cohort’s single contemporaneous measurements yielded a concordant T1 AUC (0.67 vs. 0.69, cross-validated means), suggesting this proxy did not materially distort the T1 conclusions.

Certain variables were binarized (e.g., respiratory rate below or above 22/min), while others were converted to numerical scales (e.g., SIRS criteria, ranging from 0 to 4). Both the total SOFA score and its individual component scores were included. Microbiological data captured the presence of bacterial, viral, or fungal infections as documented in clinical notes. 

Andalusian cohort: From an initial assessment of 38 different clinical–laboratory variables, only three had any missing data at all, and all were far below the 33.4% cutoff. Therefore, all 38 were included in further analyses (see Supplementary Table S2). Using these 38 variables, we performed greedy searches to identify key variables and to estimate performance. The 33.4% missing data cutoff was a pre-specified, pragmatic threshold chosen to balance imputation feasibility against variable coverage. It was not a value specifically tuned post hoc in the 510(k) cohort; rather, it was based on the level of missingness in lactate, which is considered clinically important enough to retain [1,3,16]. No Andalusian model loses more than 3.1% of patients when analyzed as complete cases (Supplementary Table S11). Jakobsen et al. propose that where the proportion of missing data is below approximately 5%, and the missingness is not concentrated in identifiable patient groups, it may be ignored and complete case analysis used as the primary analysis [14]; the complete case analyses in Supplementary Table S11 are that analysis, and they agree with the imputed results. Their guidance addresses randomized trials, so we treat the threshold as a rule of thumb rather than a formal criterion.

For both cohorts, the suspected ‘site of infection’ at time T1 refers to the attending clinician’s initial assessment and was considered to fall into one of seven mutually exclusive categories: pulmonary, abdominal, urinary, bloodstream, central nervous system (CNS), other, or ‘unknown’ (unknown or not recorded). Site of infection category ‘other’ means not classifiable in pulmonary, abdominal, urinary, CNS, or bloodstream categories. For each patient, the possibilities for site of infection were coded as either “1” (suspected) or “0” (not suspected). See Supplementary Table S1 for additional detail. 

In contrast, at time T3, the microbiological variables (confirmed bacterial/viral/fungal infection, positive blood culture, positive urine culture) represent distinct, separately coded variables derived from culture and pathogen identification data. The ‘site of infection’ variable used in the T1 analyses is not updated or revised at T3; it remains the initial clinical impression throughout all time point analyses.

### 2.4. SeptiCyte RAPID

SeptiCyte RAPID (Immunexpress Inc., Seattle, WA, USA) is a transcription-based host response test for discriminating sepsis vs. SIRS. It has received regulatory clearance for clinical use in the USA, Europe, and Australia. The test is based on quantitation of transcripts from the PLAC8 and PLA2G7 genes. The PLAC8 gene (placental associated 8) encodes a protein with diverse roles in cell proliferation, immune function, and development, including its original identification in placenta-specific tissue [17]. PLAC8 is upregulated in sepsis [18]. The PLA2G7 (phospholipase A2 Group VII) gene encodes a protein that degrades platelet-activating factor, which is a pro-inflammatory lipid [19]. PLA2G7 is downregulated in sepsis [20].

SeptiCyte RAPID is run on the Biocartis Idylla hardware platform, using version 1.2 of the test-specific software (Biocartis NV, Mechelen, Belgium). The test is performed by pipetting 0.9 mL of PAXgene-stabilized blood (corresponding to 0.24 mL of drawn blood) into a custom cartridge, which performs all assay steps including sample extraction/purification and RT-qPCR for the detection and relative quantification of the PLAC8 and PLA2G7 mRNA targets. The test has a hands-on time of ~2 min and a turnaround time of ~1 h.

Test results are calculated and presented automatically through a software-generated report, which includes a quantitative score (SeptiScore, range 0–15, higher scores correlate to increased likelihood of sepsis), calculated as the difference between the RT-qPCR Cq values for PLA2G7 and PLAC8 and proportional to sepsis probability. Four SeptiScore Bands were pre-defined in the following way, as described previously in Balk et al. (2024) [9]. An independent set of 195 patient samples that had been adjudicated by RPD was used to set the Band cutoffs. The Band 1/2 cutoff was set at 5.0 to give 90% sensitivity, and the Band 3/4 cutoff was set at 7.4 to give 80% specificity. The Band 2/3 cutoff was set at the midpoint between the two outer cutoffs. 

### 2.5. Statistical Methods

General Statistical Analyses: As a measure of test performance, we used the area under the receiver operating characteristic curve (AUC). In accordance with general practice, we consider AUC 0.80 to be the lower threshold for “good” diagnostic performance [21], as indicated by the red horizontal lines at 0.80 in Figures below. Calculations were conducted with the pROC package (version 1.19.0.1) using the methods roc or auc, implemented in R by Robin X. et al. (2011) [22]. 

Greedy search: A strategy that combines variables in order of decreasing contribution strength is referred to as a greedy search. The algorithm was implemented as described by Huang (2003) [23]. Optimal logistic combinations of variables for discriminating sepsis from SIRS in either the 510(k) or Andalusian datasets were identified using this approach. Robust variable selection was achieved through 100 repeated stratified half-sample splits (Monte Carlo cross-validation). At every iteration, the dataset is split at random into two halves, stratified on sepsis/SIRS status, one used for training and the other for testing; this is repeated 100 times. The same 100 splits are reused for every candidate variable and at every step of the search, so all candidates are compared on identical data. The halves are not swapped within a repetition, so each repetition contributes one out-of-sample estimate rather than two. The samples from the training set are not present in the test set at every iteration, and there is no overlap between the training and test sets. For every variable, at every iteration, the base generalized logistic model (GLM) is generated from the training set of data, and the AUC is estimated by predicting the performance of the base GLM on the test set of data (a new set of patients not used to generate the base GLM model). The models are of the form: y~SeptiScore, y~CRP, y~Respiratory rate (min), etc. The first feature to be selected in the new model is determined from the model having the best performance (mean AUC). This exercise is repeated to find the next best-performing variable in addition to the first variable identified up to the recursion depth specified.

Model performance was evaluated using a logistic regression decision rule, with the objective of maximizing the mean area under the receiver operating characteristic curve (AUC) across cross-validation iterations. Variables are selected using a logistic regression decision rule combined with the maximization of the mean AUC from the internal CV. The process is performed until n variables are built into the final model, with the stopping criterion of limiting the search to the total number of variables so they can be ranked by how they are incorporated into the Greedy search objectively. Greedy forward selection was chosen over alternatives such as LASSO or elastic net regularization for its tractability, interpretability, and consistency with prior biomarker discovery work in this cohort [9,11,23]. Regularized alternatives are noted as a direction for future work. Missing value imputation was performed once, across the full sample, prior to and independent of the greedy search. Only model fitting and AUC evaluation at each greedy search step were cross-validated (100 repeated stratified half-sample splits), and imputation did not use outcome (sepsis/SIRS) status. Stability of the selected variable sets was assessed by re-running the greedy search independently on 30 bootstrap resamples of each cohort’s T2 (+SeptiCyte RAPID) candidate pool (Supplementary Tables S12 and S13). Variable selection was not repeated independently within each cross-validation iteration: the cross-validation is nested inside the selection loop, scoring each candidate variable at every greedy step, and the winning variable is chosen once on the mean cross-validated AUC. Because selection and performance estimation therefore draw on the same resampling, the cross-validated AUCs are optimistic relative to a fully nested procedure and are internal validation estimates; no external validation was performed. Where several successive models in a greedy search attained the same mean AUC after rounding to two decimal places, the most parsimonious of those models was selected, and that model is the one reported throughout the manuscript and the Supplementary Materials.

Comparison of AUCs: Differences between AUCs were tested by the method of DeLong et al. [24], implemented in pROC as roc.test [22], in its paired form wherever two models were evaluated on the same patients. The AUCs obtained by refitting each infection site-independent model on its full sample (Supplementary Table S10) are apparent, in-sample values, higher than the cross-validated means on which selection was based; these tests therefore address whether two AUCs differ from each other, not whether either would replicate in new patients. Where one model’s predictors form a strict subset of another’s, DeLong’s test is conservative, because the null distribution is then non-normal [25]. Such nested comparisons arise only in the variable exclusion analyses in Supplementary Table S14, S14b and S14c, where the change in AUC rather than the *p*-value is the primary quantity; the successive time point and ±SeptiCyte RAPID comparisons are non-nested, each model having been selected by an independent greedy search. Sensitivity and specificity at each model’s Youden optimal cutoff are reported in Supplementary Table S15.

### 2.6. Assessment of Incorporation Bias

A range of clinical and laboratory variables was considered by the RPD panelists in establishing the comparator (reference) diagnoses of sepsis or SIRS. This introduced the potential for incorporation bias, since some of these same variables were later used to assess diagnostic performance. SeptiCyte RAPID and PCT, however, were not subject to incorporation bias, because the RPD panelists were blinded to these two variables. To assess the potential influence of incorporation bias, we performed a variable exclusion sensitivity analysis. A logistic regression model was constructed using seven informative variables identified by a greedy search (SeptiCyte RAPID, PCT, Tmax, RRmax, MAPmax, age, HRmin). AUCs were calculated, and AUC confidence intervals were estimated using nonparametric bootstrap resampling (2000 iterations) with percentile-based 95% intervals. We examined the change in AUC resulting from setting individual logistic regression coefficients, as well as selected combinations of coefficients, to zero. This analysis characterized the dependence of the sepsis/SIRS discrimination on each model component. Supplementary Figure S4A,B and Supplementary Table S9 present the results of these analyses. A summary of these findings is presented in Section 3.8, with implications discussed further in Section 4.4.

## 3. Results

### 3.1. Conceptual Framework for the Analysis

**Figure 1:**
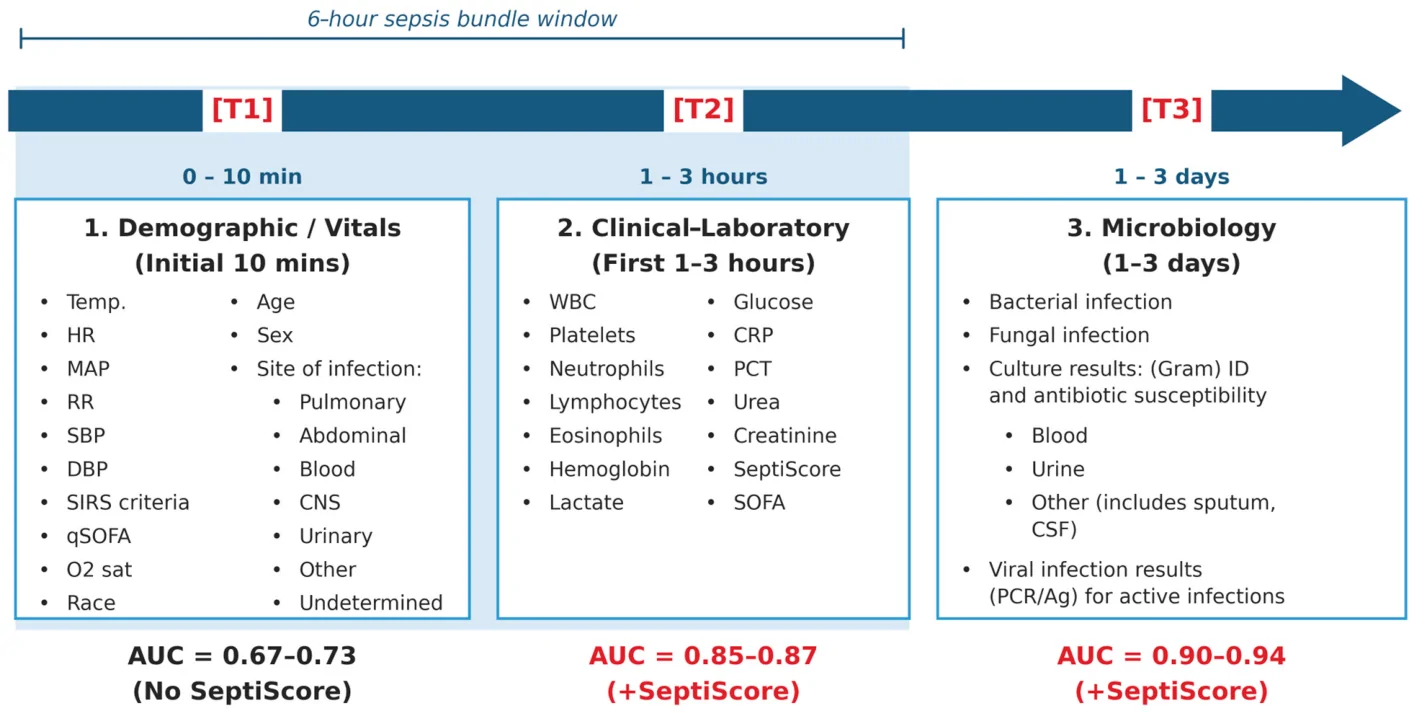
Diagnostic Variables Measured in the 510(k) and Andalusian cohorts. Variables can be classified according to their temporal availability. Time T1, immediately upon presentation: patient history, demographics, vital signs. Time T2, within 1–3 h: clinical–laboratory variables including SeptiCyte RAPID. Time T3, within 1–3 days: Microbiological culture results. Viral PCR/antigen test results, when available, were assigned to the later T3 time point because the exact timing of testing was uncertain. The mean AUC values are summarized from analyses of the 510(k) and Andalusian cohorts (Table 1 and Table 2). Abbreviations: Heart Rate (HR), Respiratory Rate (RR), Mean Arterial Pressure (MAP), Systolic Blood Pressure (SBP), Diastolic Blood Pressure (DBP), Temperature (Temp), Oxygen saturation (O2 sat), Antibiotic Susceptibility Testing (AST), Antigen (Ag), Central Nervous System (CNS), Cerebrospinal Fluid (CSF), Identification (ID), Polymerase Chain Reaction (PCR), Procalcitonin (PCT), White blood cell count (WBC), C-reactive protein (CRP), quick Sequential Organ Failure Assessment (qSOFA), Sequential Organ Failure Assessment (SOFA), Systemic Inflammatory Response Syndrome (SIRS).

Figure 1 presents a conceptual framework for our analysis, and a top-level summary of results from the independent 510(k) [9,11] and Andalusian cohorts [10]. Variables with <33.4% missing values were grouped into three categories based on their typical availability after patient presentation: patient history, demographics, and vital signs, available immediately upon presentation (time T1); clinical–laboratory variables, available within one to three hours (time T2); and microbiology variables, available within one to three days (time T3). For a full list of variables for the 510(k) and Andalusian cohorts, see Supplementary Tables S1 and S2, respectively. 

### 3.2. Analysis Based on Data from Time T1 (Patient History, Demographics, Vital Signs)

**Figure 2:**
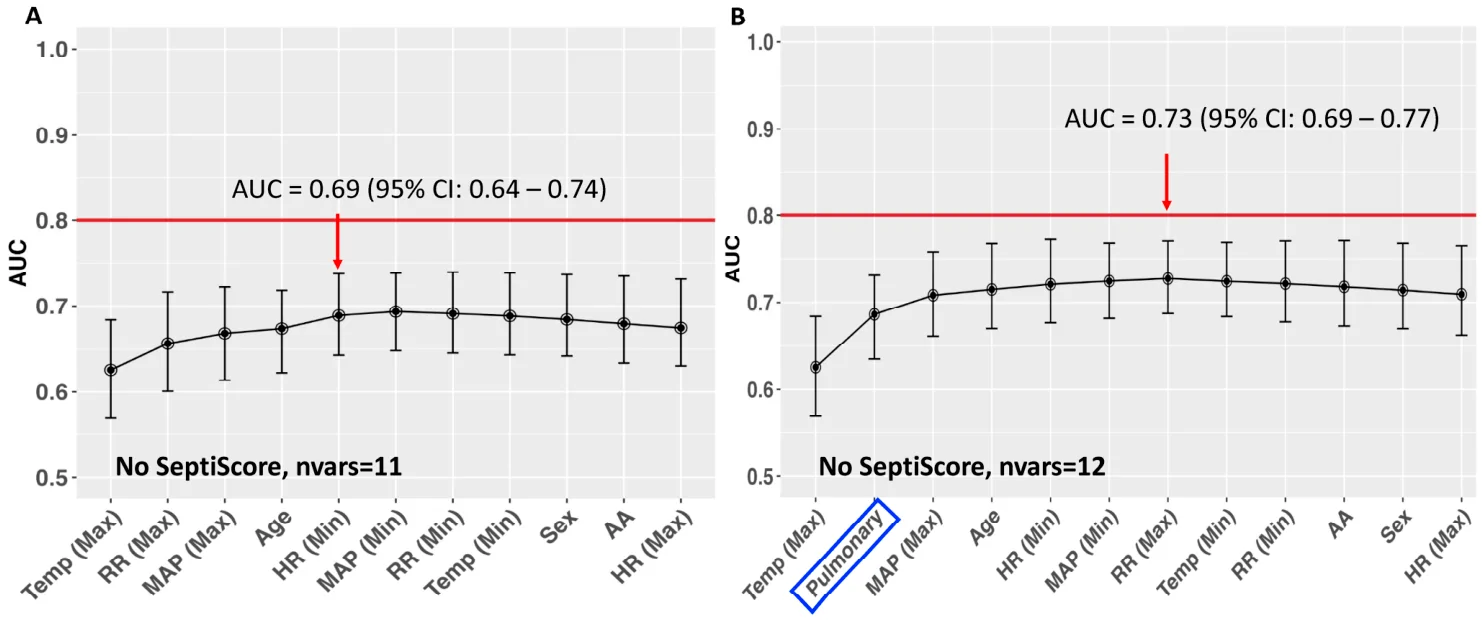
Diagnostic Performance at Time T1 (immediately upon presentation), based on patient history, demographics, vital signs. Data from the 510(k) cohort. (**A**) Site of infection not known; (**B**) Pulmonary site of infection suspected (blue box). Abbreviations: Temperature (Temp), Arterial (Art), Minimum (Min), Maximum (Max), AA (African American), Respiratory Rate (RR), Mean arterial pressure (MAP), Heart Rate (HR). Each dot and whisker represent the mean AUC ± 95% CI for the corresponding variable.

**Figure 3:**
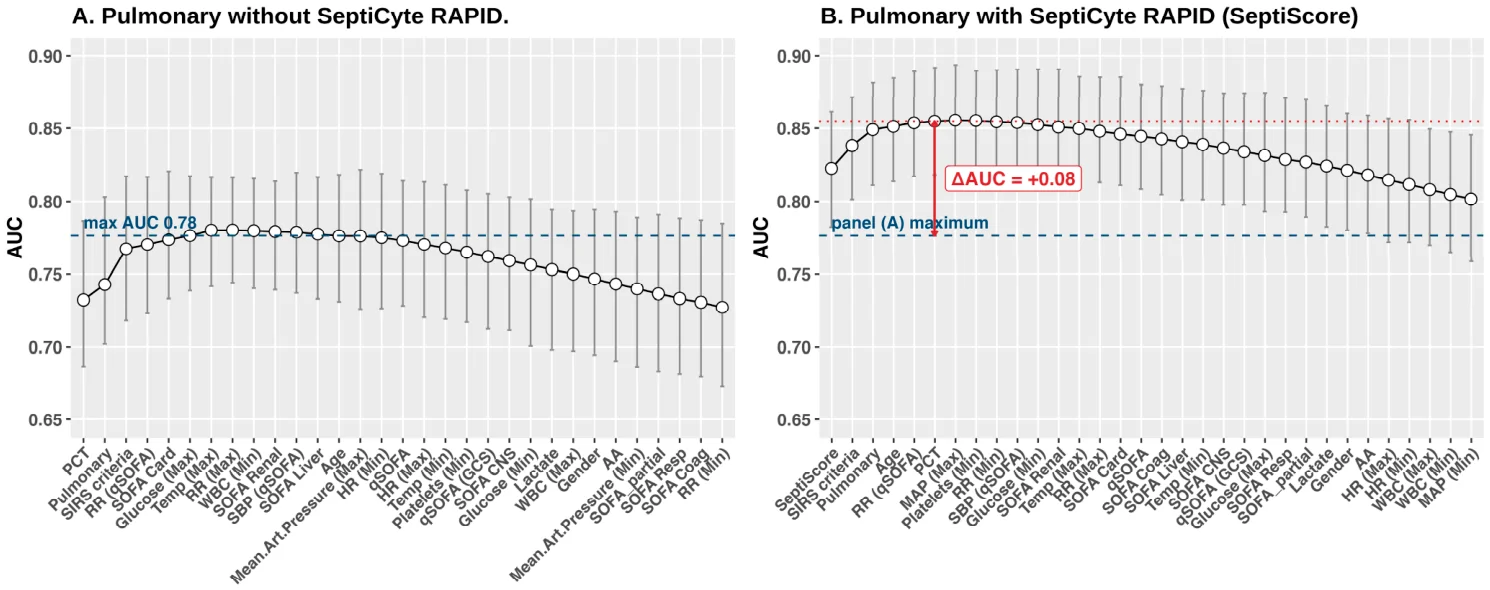
Diagnostic performance based on T1 parameters, plus clinical–laboratory test results from time T2 (1–3 h). Analysis for Pulmonary site of infection. Data from the 510(k) cohort. (**A**) Pulmonary site of infection without SeptiCyte RAPID. (**B**) Pulmonary site of infection with SeptiCyte RAPID (SeptiScore). The blue dashed line marks the highest mean AUC reached without SeptiCyte RAPID in (**A**) and is repeated in (**B**), where the arrow gives the absolute increase in AUC attributable to SeptiCyte RAPID at this site (+0.08). The red dotted line shows the maximum AUC reached after adding SeptiCyte RAPID in the combinatorial model. Comparable per-site increments are given in Table 1 and Section 3.5. Abbreviations: Procalcitonin (PCT), Systemic Inflammatory Response Syndrome (SIRS), quick Sequential Organ Failure Assessment (qSOFA), Respiratory Rate (RR), Heart Rate (HR), Temperature (Temp), Mean Arterial Pressure (MAP), Systolic blood pressure (SBP), Sequential Organ Failure Assessment (SOFA), Maximum (Max), Minimum (Min), Glasgow Coma Score (GCS), White Blood Cell Count (WBC), Respiratory (Resp), Coagulation (Coag), Arterial (Art), Cardiovascular (Card), African American (AA). Each dot and whisker represent the mean AUC ± 95% CI for the corresponding variable.

**Figure 4:**
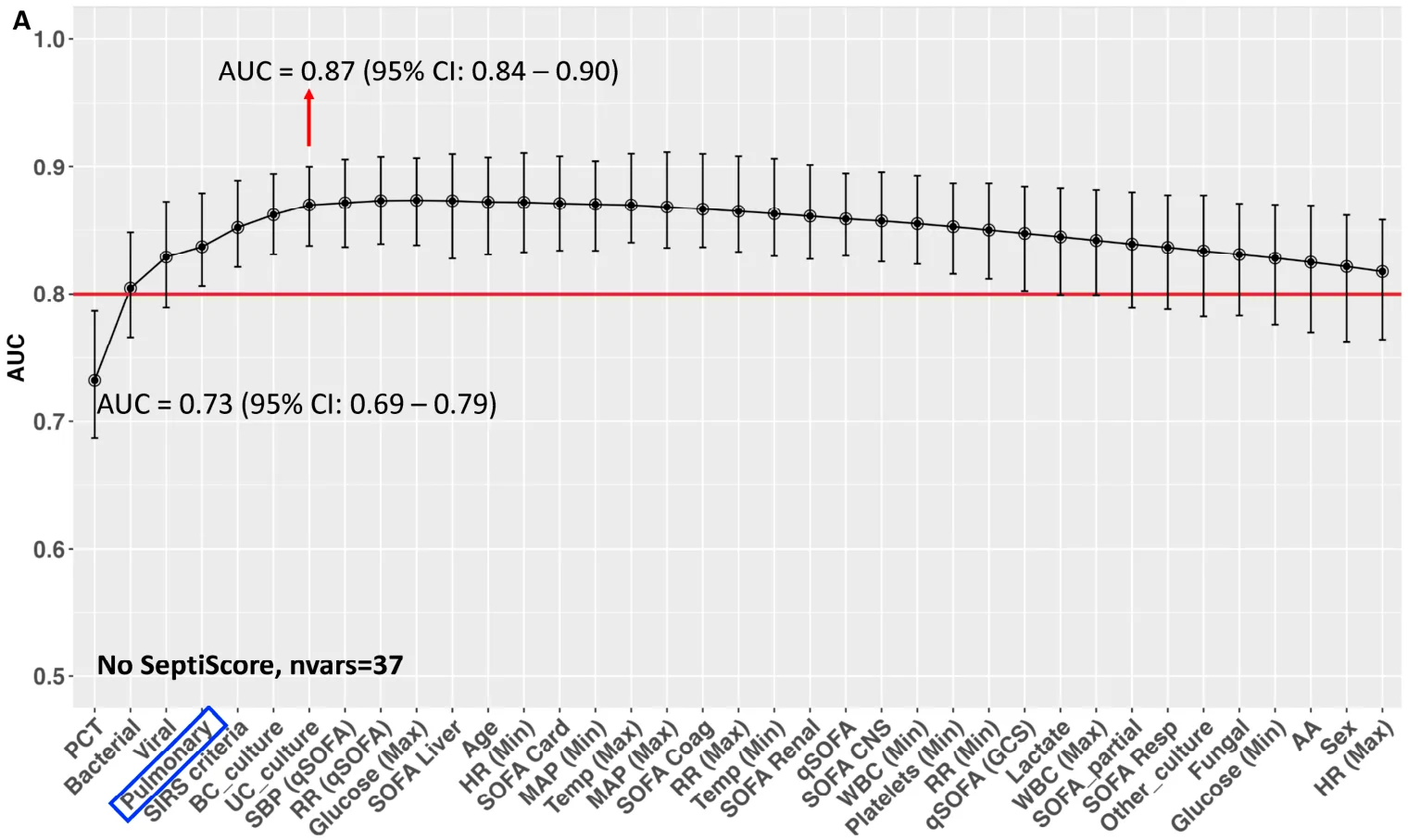

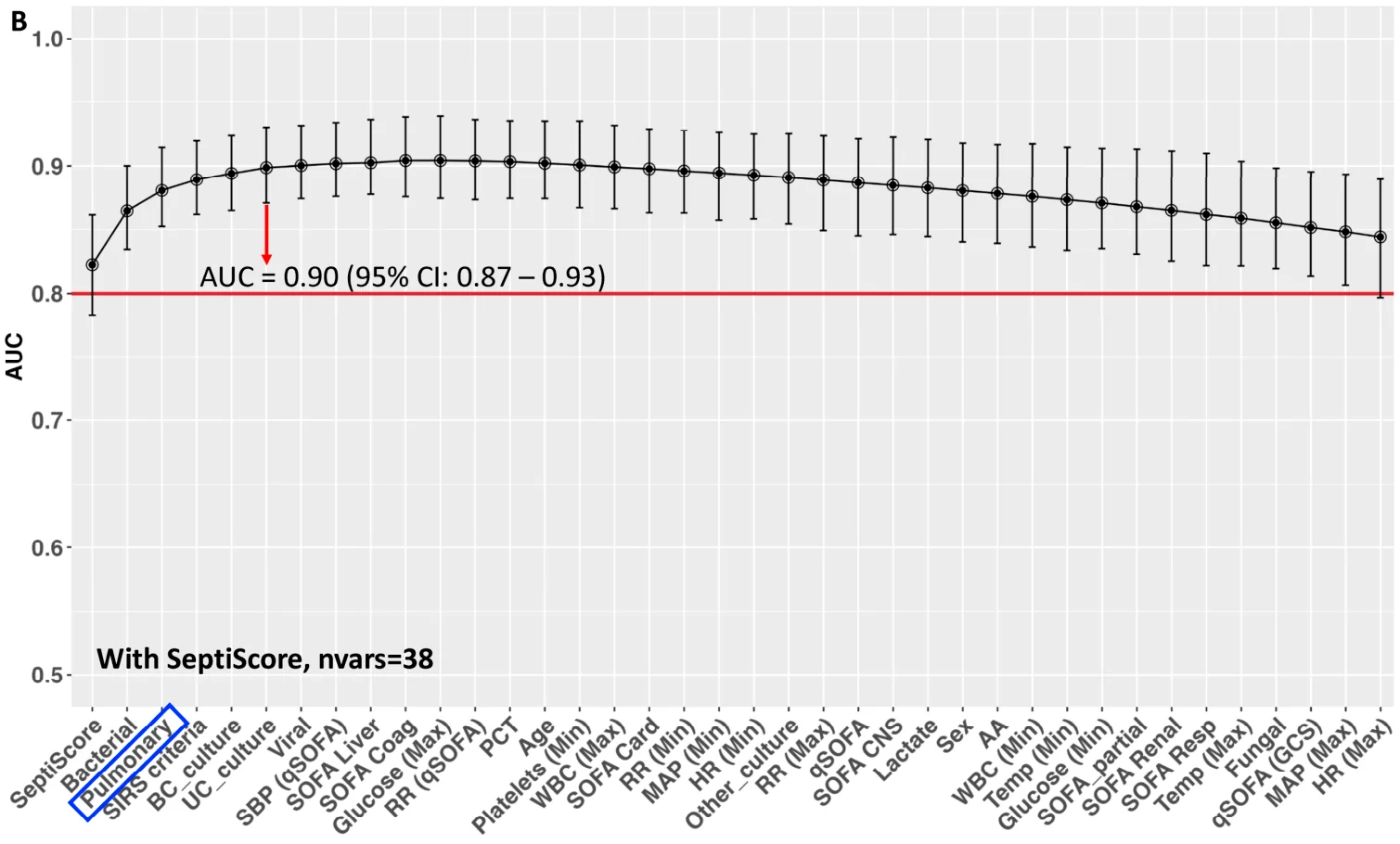
Diagnostic performance based on T1 and T2 parameters, plus microbiological culture results at time T3. Results for Pulmonary site of infection (blue box). Data from the 510(k) cohort. Greedy searches were performed (**A**) without SeptiCyte RAPID results, and (**B**) with SeptiCyte RAPID results. Abbreviations: blood culture (BC), urine culture (UC), systolic blood pressure (SBP), Procalcitonin (PCT), Systemic Inflammatory Response Syndrome (SIRS), quick Sequential Organ Failure Assessment (qSOFA), Respiratory Rate (RR), Heart Rate (HR), Temperature (Temp), Mean Arterial Pressure (MAP), Sequential Organ Failure Assessment (SOFA), Maximum (Max), Minimum (Min), Glasgow Coma Score (GCS), White Blood Cell Count (WBC), Respiratory (Resp), Coagulation (Coag), Arterial (Art), Cardiovascular (Card), African American (AA). Each dot and whisker represent the mean AUC ± 95% CI for the corresponding variable.

At time T1, we performed greedy searches on the variables available immediately upon presentation; Figure 2, Figure 3 and Figure 4 illustrate results for a suspected pulmonary site of infection, the most common site among patients retrospectively determined to have sepsis [16]. Figure 2 shows the searches when the site of infection was not known (A) and when a pulmonary site was suspected (B): the maximum AUC was 0.69 (95% CI: 0.64–0.74) using five variables and 0.73 (95% CI: 0.69–0.77) using seven, with other suspected sites falling in the same 0.69–0.73 range (Supplementary Figure S1A–F). Temperature (max) was the single most important contributor to AUC across all searches. Where the site of infection was known, it was the second most important contributor, adding approximately 0.04 AUC units. The remaining contributing variables, and the order in which they were selected for each suspected site, are shown in Figure 2.

### 3.3. Addition of Clinical–Laboratory Data (Time T2: 1–3 H)

For evaluation at one to three hours, a large number of clinical–laboratory variables became available. Figure 3 shows the greedy search results for a pulmonary site of infection, when SeptiCyte RAPID results were excluded (panel A) or included (panel B). The dashed line in both panels marks the maximum AUC reached without SeptiCyte RAPID, so that the increase attributable to SeptiCyte RAPID can be read directly from panel B. Plots for all other suspected sites of infection are included in the Supplementary Materials.

When excluding SeptiCyte RAPID, the maximum AUC was 0.78 (95% CI: 0.74–0.82), which was achieved with six variables (Figure 3A). The variables that consistently contributed the most to AUC across all searches were PCT, site of infection, and SIRS criteria. Procalcitonin (PCT) was the single most important variable across all greedy searches when SeptiCyte RAPID was not included. The absolute improvement in AUC attributable to SeptiCyte RAPID at this step was +0.08 (0.86 vs. 0.78); comparable per-site deltas are summarized in Section 3.5.

When including SeptiCyte RAPID, the maximum AUC was 0.86 (95% CI: 0.82–0.89), which was achieved with six variables (Figure 3B). The variables that consistently contributed the most to AUC across all searches were SeptiScore, site of infection, and SIRS criteria. SeptiCyte RAPID was the single most important variable that contributed to AUC across all greedy searches and was not synergistic with PCT. The AUC gain from combining SeptiCyte RAPID with PCT did not exceed SeptiCyte RAPID’s individual contribution (Supplementary Table S9: removing PCT from a SeptiCyte-containing model changed AUC by only −0.01).

### 3.4. Addition of Microbiological Culture Results (Time T3: 1–3 Days)

For assessment at one to three days, microbiological culture results became available. Figure 4 summarizes the diagnostic performance that could be obtained by combining test results from T1 (time of presentation), T2 (clinical–laboratory data at one to three hours), and T3 (microbiological culture results at one to three days). Without SeptiCyte RAPID, the AUC could be increased to 0.87 (95% CI: 0.84–0.90) using seven variables (panel A). With SeptiCyte RAPID, the AUC could be increased to 0.90 (95% CI: 0.87–0.93) with six variables (panel B).

Key variables driving performance included SeptiCyte RAPID, PCT, confirmed bacterial or viral infection, site of infection, and positive culture results. After the microbiological culture data from time T3 became available, the additional performance boost from including SeptiCyte RAPID was small (0.90–0.87 = 0.03 AUC units). However, because treatment for sepsis is such a time-critical problem, the benefit of waiting one to three days for culture results is outweighed by the risk of not treating a patient immediately.

### 3.5. Comparison Across Different Suspected Sites of Infection

Greedy search analyses were also performed on the 510(k) dataset, after incorporating information on suspected or presumed sites of infection. Within a particular timeframe of analysis (T1, T2, or T3), the AUCs did not vary significantly between suspected infection sites; see Supplementary Figures S1A–F, S2A–F, and S3A–F, respectively.

### 3.6. Analysis of Data from the Andalusia Cohort

We conducted comparable greedy search analyses on the data from the Andalusian cohort. AUCs for the Andalusian cohort at successive time points were comparable to AUCs from the 510(k) cohort, and similarly increased as more diagnostic information became available over time (see Table 2). As in the 510(k) dataset, AUCs in the Andalusian dataset did not vary significantly by the suspected/presumed site of infection, for any of the time-delimited analyses (T1, T2, or T3), see Supplementary Tables S4–S8.

### 3.7. Comparison of Analysis Results from the 510(k) and Andalusian Cohorts

It was not possible to formally cross-validate the results from the 510(k) and Andalusian cohorts, because the variables measured and analyzed in the two cohorts were not exactly the same. Nonetheless, there was a significant overlap of approximately 28 variables (see Supplementary Table S3). While variations in clinical practice from site to site are to be expected, the fact that many of the same variables were tested at different sites, under different study protocols, increases confidence in the generality of the findings described here. Comparing the two sets of analyses, we conclude the following:

I. At time T1 (presentation), regardless of the combination of variables based on patient history, demographics, and vital signs, the maximum AUC that could be obtained was around 0.70 ± 0.03.
II. At time T2 (one to three hours after presentation), the addition of clinical–laboratory variables boosted the AUC to 0.85 ± 0.02. SeptiCyte RAPID test results became available at this time, and the SeptiScore had the greatest positive impact on AUC of all variables available at that time.

510(k) cohort (Table 1): ΔAUC values are obtained by subtracting the performance when SeptiCyte is not included from when it is included (e.g., Pulmonary: 0.86–0.78 = +0.08; Abdominal: 0.86–0.78 = +0.08; CNS: 0.85–0.76 = +0.09; None: 0.85–0.76 = +0.09). Thus, with the addition of SeptiCyte RAPID at T2, the ΔAUC ranges from +0.08 to +0.09 across all sites of infection.

Andalusian cohort (Table 2): ΔAUC values are obtained by subtracting the performance when SeptiCyte is not included from when it is included (e.g., Pulmonary: 0.87–0.80 = +0.07; Other: 0.87–0.81 = +0.06). Thus, with the addition of SeptiCyte RAPID at T2, the ΔAUC ranges from +0.06 to +0.07 across all sites of infection.

I. At time T3 (one to three days after presentation), yet another performance gain was achieved through incorporation of positive microbiological culture results and pathogen identification, boosting the AUC further to 0.86–0.91 without SeptiCyte RAPID, or to AUC 0.90–0.94 with SeptiCyte RAPID. While unsurprising, the microbiological culture and pathogen ID data became available only after the critical early treatment window (T2, one to three hours after presentation), when timely initiation of appropriate sepsis therapy is most consequential.
II. In logistic regression, the performance benefit obtained by combining variables typically plateaus after approximately four to seven variables, after which model performance may begin to decline. This well-recognized phenomenon likely reflects several contributing factors, including sensitivity of the logistic regression model to parameter estimates near the decision boundary, incorporation of noise from variables weakly associated with outcomes, overfitting arising from fitting an increasing number of parameters to a fixed dataset, and redundancy introduced by highly correlated predictor variables [26].

### 3.8. Analysis of Potential Incorporation Bias

The present analysis suffers to some extent from the possibility of incorporation bias, because some of the variables used in performance calculations were also used in the reference adjudication process. We have addressed this concern in the following way. A logistic regression model using seven informative variables, identified by greedy feature selection (SeptiCyte RAPID, PCT, temperature (max), RR (max), MAP (max), age, HR (min)), yielded an AUC of 0.85 (95% CI: 0.80–0.89). A model incorporating only SeptiCyte RAPID and PCT, variables not used by the RPD panel and therefore not subject to incorporation bias, yielded an AUC of 0.83 (95% CI: 0.78–0.87) (See Supplementary Figure S4B and Table S9). Therefore, the majority of the model’s discriminative performance was preserved using variables independent of the RPD. It appears that the variables temperature (max), RR (max), MAP (max), age, and HR (min) together contribute only +0.02 units to the overall AUC performance measure. A model excluding just SeptiCyte RAPID yielded an AUC of 0.75 (95% CI: 0.70–0.79) (See Supplementary Figure S4A and Table S9). Exclusion of SeptiCyte RAPID produced the largest reduction in AUC (Δ AUC = −0.10), while exclusion of the remaining variables resulted in smaller changes (|Δ AUC| < 0.01). No single variable subject to incorporation bias accounted for more than 0.01 AUC units. We conclude that, in our analysis, incorporation bias exerts a relatively small effect on AUC-based performance estimates. The same variable exclusion logic was applied to the Andalusian cohort (Supplementary Table S14). At T2, the full four-variable model reached an AUC of 0.88. In comparison, SeptiCyte RAPID alone accounted for an AUC of 0.84, and the clinical variables alone gave an AUC of 0.67. At T3, the full model reached an AUC of 0.93, SeptiCyte RAPID alone at 0.84, and the microbiological culture variables alone gave an AUC of 0.81. The latter variables were bacterial pathogen, positive blood culture, positive urine culture, and fungal pathogen, all of which were available to the Andalusian adjudicators. Incorporation bias is therefore more material in the Andalusian cohort than in the 510(k) cohort, and the Andalusian T3 estimate in particular should be read as an upper bound.

## 4. Discussion

We attempted to quantify the diagnostic value of data collected at different times during a patient’s transit through the hospital, for diagnosing sepsis vs. SIRS. Data from two independent study cohorts (510(k), Andalusia) were examined, with similar results. The 510(k) cohort consisted of 419 patients, and the Andalusian cohort consisted of 353 patients. Both cohorts were composed of critically ill patients in the ICU and were retrospectively diagnosed with either sepsis or SIRS. Although it was not possible to pool the data from the two cohorts (because not all variables were common), similar results were obtained in the two cases, supporting the general approach proposed here.

Diagnostic variables were stratified by their temporal availability to represent the evolving clinical picture within the first three days from hospital admission to ICU. Three time points were considered: T1, immediately upon presentation; T2, within one to three hours when clinical–laboratory results became available; and T3, within one to three days when microbiological culture results became available. The analysis revealed that diagnostic performance, measured by AUC, increased progressively from ~0.7 using clinical variables to ~0.9 when incorporating all variables, including microbiological culture and SeptiCyte RAPID results. The greatest single time point gain in AUC (from ~0.7 to ~0.85) occurred with the inclusion of SeptiCyte RAPID at time T2. Additional clinical and laboratory measures did not add much more diagnostic value until time T3, when blood culture results became available, which boosted the AUC performance up to ~0.9. 

This study therefore demonstrates that diagnostic performance at time T1 is limited to AUC ~0.7 using just the data available at presentation (patient history, demographics, and vital signs). However, an efficient diagnostic workup at time T2 (one to three hours after presentation) can substantially improve the accuracy of identifying sepsis in critically ill patients suspected of sepsis. Specific clinical–laboratory variables available at T2 can be used to accurately identify patients with sepsis in lieu of traditional microbiological culture results. We argue that in most patients suspected of sepsis, the results available at T2 are accurate and timely enough to make informed treatment and downstream diagnostic decisions.

### 4.1. Upon Presentation (Time T1)

At presentation (time T1), a limited set of readily measured variables, including vital signs such as maximum temperature, mean arterial pressure (MAP), respiratory rate, and suspected site of infection, provided modest discriminatory power (AUC = 0.69–0.73). This modest discriminatory power is similar to that previously reported in the literature when using SOFA (0.74) and qSOFA (0.73) variables [27]. These findings, although limited in terms of AUC, reinforce the importance of prompt clinical assessment and vital sign interpretation, which remain cornerstones of early sepsis recognition [3]. Interestingly, respiratory rate emerged as a key variable contributing to AUC except in respiratory infections, reflecting its general association with systemic illness rather than site-specific infection. These variables overlap substantially with components of early warning scores such as SIRS, NEWS2, MEWS, and qSOFA [28], though qSOFA itself showed limited predictive enhancement in this cohort, aligning with recent critiques of its diagnostic utility [29]. Temperature behaved as the most informative single vital sign at this time point: removing maximum temperature from the T1 model cost 0.05 AUC units, more than any other variable (Supplementary Table S14c). This is consistent with emergency department data in which the best clinical model for identifying an infectious etiology among patients with abnormal vital signs retained initial temperature as an independent predictor (odds ratio 1.6, 95% CI 1.1–2.2) and reached an AUC of 0.76 [30].

### 4.2. Assessment with Clinical–Laboratory Tests (Time T2)

At time T2 (one to three hours), clinical–laboratory test results became available and could be added to the diagnostic workup. Diagnostic accuracy improved accordingly (AUC~0.77). When SeptiCyte RAPID results were included, performance increased to AUC~0.85, demonstrating its superior discriminatory performance compared to procalcitonin (PCT) and lactate. PCT, while commonly used, neither outperformed nor acted synergistically with SeptiCyte RAPID, underscoring the latter’s value as an independent biomarker of host response to sepsis, rather than just the presence of a bacterial infection. These findings are consistent with prior studies showing the diagnostic challenges posed by overlapping inflammatory responses and the need for molecular host response assays to reduce diagnostic uncertainty [2,31]. 

### 4.3. Microbiology Assessment (Time T3)

By one to three days post-presentation (Time T3), the incorporation of microbiological culture results further increased AUC to ~0.9, particularly when combined with the SeptiCyte RAPID scores. Culture results added approximately 0.04–0.07 AUC units of diagnostic gain when SeptiCyte data were already available, reflecting the limited incremental value of delayed microbiological confirmation in guiding early therapy. This suggests that rapid, molecular-based diagnostics may provide clinicians with most of the actionable information needed within the first few hours, a timeframe critical for meeting sepsis management guidelines [32]. Because adjudicators in both cohorts had access to microbiological results, these T3 estimates should be interpreted as upper bounds rather than unbiased estimates, more so for the Andalusian cohort, where the microbiology variables alone reach an AUC of 0.81 against a full-model AUC of 0.93 (Supplementary Table S14).

### 4.4. Study Limitations

There are several limitations to this study. First, the datasets, though multicentric and demographically diverse, included only ICU patients and may not generalize to non-ICU populations, such as emergency departments or wards. Nonetheless, the consistently high performance of SeptiCyte RAPID across infection sites supports its robustness as an adjunct to clinical judgment. A second limitation is that clinical–laboratory variables were not consistently collected across the two cohorts, making it challenging to determine the commonality of variables that contributed to diagnostic AUC. More extensive studies using more heterogeneous patient populations and common variables may be required to confirm the results presented here. Third, this retrospective analysis is subject to some degree of incorporation bias, because a number of variables evaluated for performance were also used by the physician adjudication panel to establish the reference standard. This overlap likely inflates the apparent performance of models incorporating these variables. Incorporation bias is a well-recognized limitation in retrospective studies, particularly those relying on physician adjudication [33,34,35]. Our study design accounted for this by specifically blinding the RPD panel members to SeptiCyte RAPID and PCT, which therefore are not susceptible to this particular source of bias. Our further analyses (Supplementary Figure S4A,B and Table S9) suggested that the inflationary effect of incorporation bias in this study was limited to only about +0.02 AUC units. More fundamentally, the reference diagnoses in both cohorts are retrospective, physician-adjudicated classifications rather than an objective gold standard, and all AUCs reported here should be interpreted with that caveat. A related limitation is that, for the 510(k) cohort, the maximum and minimum values recorded within 24 h of ICU admission were used as a proxy for single values at presentation, consistent with the convention used for APACHE II and SOFA scoring, whereas the Andalusian cohort contributed single contemporaneous measurements. A further limitation concerns the cross-validation procedure: missing value imputation was performed once, across the full dataset, rather than being nested within each cross-validation fold, which might introduce a bias. Simple mean imputation of missing values was used for data analysis. The complete case sensitivity analysis in Supplementary Table S11 indicates that this did not materially alter discrimination in any of the five models in either cohort. That analysis speaks only to the AUC point estimates, however: because each imputed value is treated as though it had been observed, the confidence intervals reported here do not reflect the additional uncertainty that single imputation introduces and are correspondingly narrower than multiple imputation would yield. Likelihood ratios for SeptiScore itself, and the post-test probabilities that follow from them, have recently been reported by our group [36]. In a retrospective analysis of 889 critically ill patients recruited across 19 hospitals in the USA and Europe, band-specific likelihood ratios were derived for SeptiScore in the discrimination of sepsis from non-infectious SIRS: the median LR+ was 3.26 (range 2.57–4.24) for Band 3 and 6.97 (range 4.35–15.57) for Band 4, and the median LR− was 0.16 (range 0.14–0.20) for Band 2 and 0.085 (range 0.014–0.16) for Band 1. These are the quantities a practitioner would combine with their own pretest estimate to obtain an individualized, patient-specific post-test probability of sepsis [37]. Finally, the T1/T2/T3 windows were fixed by data availability rather than by a clinical bundle rationale, and neither cohort recorded laboratory timestamps granular enough to reconstruct alternative windows; clinically motivated alternatives such as a six-hour bundle window merit evaluation in future studies with more granular timestamped data.

### 4.5. Clinical Utility Context

Implementing time-based diagnostic frameworks that integrate rapid molecular tests (pathogen- and host response-based) may optimize both antimicrobial stewardship and patient outcomes. Ultimately, even with complete data, diagnostic accuracy reached a maximum AUC around 0.90, emphasizing the intrinsic complexity of sepsis diagnosis [13], the lack of a true gold standard for sepsis diagnosis [38], and the continuing need for integrated, multimodal approaches [39].

The results of this analysis align with calls to refine sepsis bundles to balance speed and diagnostic precision [3,40,41,42]. That is, not all patients suspected of sepsis need sepsis bundles within certain timeframes, and flexibility with respect to timing and bundle use/compliance leads to better outcomes. Therefore, rather than rigid bundles for sepsis, it has been recommended that clinicians focus on a “time-limited course of rapid investigation” (Surviving Sepsis Campaign, 2026 Guidelines Adults) [3] or place an emphasis on “diagnostic strategies and outcomes” [42].

The long-term consequences of sepsis extend well beyond the acute episode, underscoring the importance of early and accurate diagnosis. Patients who develop chronic critical illness (CCI) following sepsis, defined as persistent organ dysfunction requiring intensive care for ≥14 days, face poor long-term functional outcomes regardless of the source of sepsis. The evidence suggests that reversing established organ dysfunction is far more challenging than preventing it at an earlier stage [43]. Furthermore, the diagnostic difficulty of sepsis is so fundamental that it persists even after death: autopsy-based findings in sepsis are largely non-specific, and specialized immunohistochemical biomarker approaches are required to confirm sepsis diagnosis post-mortem [44]. Together, these observations reinforce the clinical rationale for the time-stratified diagnostic framework presented here: earlier and more accurate real-time identification of sepsis, facilitated by host response testing such as SeptiCyte RAPID, has the potential to prevent the downstream progression to CCI and to reduce the burden of diagnostic uncertainty that currently spans the entire clinical encounter.

## 5. Conclusions

We have shown that SeptiCyte RAPID provides valuable information as part of a diagnostic workup and within a limited timeframe. Its use, in combination with other parameters and clinical judgment, may lead to more judicious use of antibiotics, fewer missed diagnoses of “sepsis mimics” and appropriate implementation of sepsis bundles in patients suspected of sepsis who actually have a higher likelihood of sepsis. This study was concerned with the temporal dependence of availability of diagnostic information; it did not assess clinical outcomes or antimicrobial stewardship performance directly, and prospective studies are needed to determine whether the observed gains in diagnostic accuracy translate into improved patient outcomes.

## Figures and Tables

**Table 1:** Summary of Performance (mean AUC of best model ± 95% CI) at Three Time Points (T1, T2 or T3), for patients stratified by sites of infection. Data from the 510(k) cohort.

	Suspected Site of Infection							
Time Point (T1, T2 or T3)	Pulmonary	Abdominal	Blood	Urinary	CNS	Other	None	Mean of Best-Model’s AUC (95% CI)
Vitals and Demographics data only (T1)	0.73 (0.69–0.77)	0.73 (0.67–0.78)	0.71 (0.66–0.76)	0.73 (0.68–0.77)	0.69 (0.64–0.74)	0.70 (0.65–0.74)	0.69 (0.64–0.74)	0.71 (0.66–0.76)
Clinical–Laboratory data without SeptiCyte (T2)	0.78 (0.74–0.82)	0.78 (0.73–0.83)	0.77 (0.72–0.81)	0.78 (0.73–0.82)	0.76 (0.71–0.80)	0.77 (0.72–0.81)	0.76 (0.71–0.80)	0.77 (0.72–0.81)
Clinical–Laboratory data with SeptiCyte (T2)	0.86 (0.82–0.89)	0.86 (0.82–0.89)	0.86 (0.82–0.90)	0.86 (0.82–0.89)	0.85 (0.80–0.89)	0.85 (0.81–0.88)	0.85 (0.80–0.89)	0.86 (0.81–0.89)
Microbiology culture data without SeptiCyte (T3)	0.87 (0.84–0.90)	0.88 (0.84–0.91)	0.87 (0.83–0.90)	0.87 (0.83–0.90)	0.86 (0.82–0.89)	0.86 (0.82–0.89)	0.86 (0.82–0.89)	0.87 (0.83–0.90)
Microbiology culture data with SeptiCyte (T3)	0.90 (0.87–0.93)	0.91 (0.88–0.94)	0.90 (0.87–0.93)	0.90 (0.87–0.92)	0.90 (0.86–0.93)	0.90 (0.86–0.93)	0.90 (0.86–0.93)	0.90 (0.87–0.93)

**Table 2:** Summary of Performance (mean AUC of best model ± 95% CI) at Three Time Points (T1, T2 or T3), for patients stratified by sites of infection. Data from the Andalusian cohort.

	Suspected Site of Infection							
Time Point (T1, T2 or T3)	Pulmonary	Abdominal	Blood	Urinary	CNS	Other	None	Mean of Best-Model’s AUC (95% CI)
Vitals and Demographics data only (T1)	0.67 (0.62–0.74)	0.67 (0.62–0.74)	0.68 (0.63–0.74)	0.67 (0.62–0.74)	0.67 (0.62–0.74)	0.68 (0.63–0.74)	0.67 (0.62–0.74)	0.67 (0.62–0.74)
Clinical–Laboratory data without SeptiCyte (T2)	0.8 (0.75–0.85)	0.8 (0.75–0.85)	0.8 (0.75–0.85)	0.8 (0.75–0.85)	0.8 (0.75–0.85)	0.81 (0.76–0.86)	0.8 (0.75–0.85)	0.80 (0.75–0.85)
Clinical–Laboratory data with SeptiCyte (T2)	0.87 (0.83–0.91)	0.87 (0.83–0.91)	0.87 (0.83–0.91)	0.87 (0.83–0.91)	0.87 (0.83–0.91)	0.87 (0.83–0.91)	0.87 (0.83–0.91)	0.87 (0.82–0.91)
Microbiology culture data without SeptiCyte (T3)	0.9 (0.86–0.92)	0.9 (0.86–0.92)	0.9 (0.86–0.92)	0.9 (0.86–0.92)	0.9 (0.86–0.92)	0.91 (0.88–0.93)	0.9 (0.86–0.92)	0.90 (0.87–0.93)
Microbiology culture data with SeptiCyte (T3)	0.93 (0.9–0.95)	0.93 (0.9–0.95)	0.93 (0.9–0.95)	0.93 (0.9–0.95)	0.93 (0.9–0.95)	0.94 (0.91–0.96)	0.93 (0.9–0.95)	0.93 (0.90–0.95)

## Data Availability

The datasets presented in this article are not readily available for patient privacy and commercial reasons. Requests to access the datasets should be directed to Richard Brandon.

## Supplementary Materials

The following supporting information can be downloaded at: https://www.mdpi.com/article/10.3390/diseases14090319/s1, Table S1. List of Variables and Missing Data Proportions in the 510(k) cohort. Table S2. List of Variables and Missing Data Proportions in the Andalusian cohort. Table S3. Overlap of Variables Between Analyses of 510(k) and Andalusian cohorts. Figure S1. (A–F). Greedy search plots showing performance (AUC) of various clinical variables available at time T1 (presentation), for various initially suspected sites of infection. Figure S2. (A–F). Greedy search plots showing performance (AUC) of various Clinical–Laboratory variables available at time T2 (within 1–3 h), for various initially suspected sites of infection. Figure S3. (A–F). Greedy search plots showing performance (AUC) of various clinical variables available at Time T3 (within 1–3 days), for various initially suspected sites of infection. Table S4. Greedy Search Results for the Andalusian cohort, at Time T1 (Presentation). Table S5. Greedy Search Results for the Andalusian cohort at Time T2 (1–3 h Post-Presentation) Without SeptiCyte RAPID. Table S6. Greedy Search Results for the Andalusian cohort at Time T2 (1–3 Hours Post-Presentation) With SeptiCyte RAPID. Table S7. Greedy Search Results for the Andalusian cohort at Time T3 (1–3 Days Post-Presentation) Without SeptiCyte RAPID. Table S8. Greedy Search Results for the Andalusian cohort at Time T3 (1–3 Days Post-Presentation) With SeptiCyte RAPID. Figure S4. Investigation of potential incorporation bias by comparative ROC curve analysis. Table S9. Relative magnitude of contributions of logistic regression input variables, arranged in decreasing order of impact upon output AUC. Table S10. DeLong tests comparing AUC across time points, both cohorts. Table S11. Complete case sensitivity analysis, all five models in both cohorts. Table S11b. Study composition of the 510(k) complete case subset. Table S11c. Differential-attenuation check: SeptiCyte RAPID increment re-estimated with no imputed values in either arm. Table S12. Variable-selection stability, 30 bootstrap resamples (Andalusian cohort). Table S13. Variable-selection stability, 30 bootstrap resamples (510(k) cohort). Table S14. Incorporation-bias variable exclusion analysis extended to the Andalusian T2/T3 and 510(k) T1/T3 models. Table S14b. 510(k) T3 leave-one-out detail. Table S14c. 510(k) T1 leave-one-out detail. Table S15. Sensitivity and specificity of the infection site-independent models at their Youden-optimal cutoffs, both cohorts [45].

## Abbreviations

The following abbreviations are used in this manuscript:

Ag/Ab	Antigen/Antibody (testing)
AST	Antibiotic Susceptibility Testing
AUC	Area Under the Receiver Operating Characteristic Curve
CNS	Central Nervous System
DBP	Diastolic Blood Pressure
HR	Heart Rate
ICU	Intensive Care Unit
ID	Identification
MAP	Mean Arterial Pressure
MEWS	Modified Early Warning Score
NEWS	National Early Warning Score
OECD	Organization for Economic Co-operation and Development
PCT	Procalcitonin
PLA2G7	Phospholipase A2 Group 7 (gene)
PLAC8	Placenta-specific 8 (gene)
qSOFA	Quick Sequential Organ Failure Assessment
RPD	Retrospective Physician Diagnosis
RR	Respiratory Rate
RT-qPCR	Reverse Transcriptase quantitative Polymerase Chain Reaction
SBP	Systolic Blood Pressure
SEP-1	Severe Sepsis and Septic Shock Early Management Bundle (USA)
SIRS	Systemic Inflammatory Response Syndrome
SOFA	Sequential Organ Failure Assessment
T max	Maximum body temperature in a 24 h period
USC	University of Southern California
WBC	White Blood Cell (Count)
