# Supplementary material for: Sequential Application of Time-Stratified Demographic, Vital, Clinical–Laboratory, and Microbiology Variables for Accurate and Rapid Identification of Sepsis
Source: Diseases. 2026 Sep 1;14(9):319. doi: 10.3390/diseases14090319 (PMC13605279; doi:10.3390/diseases14090319)
Supplement: Supplementary file 1 [file diseases-14-00319-s001.zip › diseases-4389552-supplementary.pdf]

Sequential application of time-stratified demographic, vital, clinical-laboratory and microbiology variables for accurate and rapid identification of sepsis

SUPPLEMENTARY MATERIALS

Table S1. List of Variables and Missing Data Proportions in the 510k cohort

Table S2. List of Variables and Missing Data Proportions in the Andalusian cohort

Table S3. Overlap of Variables Between Analyses of 510k and Andalusian Cohorts

Figure S1 (A-F). Greedy search plots showing performance (AUC) of various clinical variables available at time T1 (presentation), for various initially suspected sites of infection.

Figure S2 (A-F). Greedy search plots showing performance (AUC) of various clinical laboratory variables available at time T2 (within 1-3 hours), for various initially suspected sites of infection.

Figure S3 (A-F). Greedy search plots showing performance (AUC) of various clinical variables available at Time T3 (within 1-3 days), for various initially suspected sites of infection.

Table S4. Greedy Search Results for the Andalusian Cohort, at Time T1 (Presentation).

Table S5. Greedy Search Results for the Andalusian Cohort at Time T2 (1-3 Hours Post-Presentation) Without SeptiCyte RAPID.

Table S6. Greedy Search Results for the Andalusian Cohort at Time T2 (1-3 Hours Post-Presentation) With SeptiCyte RAPID.

Table S7. Greedy Search Results for the Andalusian Cohort at Time T3 (1-3 Days Post-Presentation) Without SeptiCyte RAPID.

Table S8. Greedy Search Results for the Andalusian Cohort at Time T3 (1-3 Days Post-Presentation) With SeptiCyte RAPID.

Figure S4. Investigation of potential incorporation bias, by comparative ROC curve analysis.

Table S9. Relative magnitude of contributions of logistic regression input variables, arranged in decreasing order of impact upon output AUC.

Table S10. DeLong tests comparing AUC across time points, both cohorts.

Table S11. Complete-case sensitivity analysis, all five models in both cohorts.

Table S11b. Study composition of the 510k complete-case subset.

Table S11c. Differential-attenuation check: SeptiCyte RAPID increment re-estimated with no imputed values in either arm.

Table S12. Variable-selection stability, 30 bootstrap resamples (Andalusian cohort, T2+SeptiCyte pool).

Table S13. Variable-selection stability, 30 bootstrap resamples (510k cohort, T2+SeptiCyte pool).

Table S14. Incorporation-bias variable-exclusion analysis extended to the Andalusian T2/T3 and 510k T1/T3 models.

Table S14b. 510k T3 leave-one-out detail.

Table S14c. 510k T1 leave-one-out detail.

Table S15. Sensitivity and specificity of the infection site independent models at their Youden-optimal cutoffs, both cohorts.

Supplementary Table S1. List of Variables and Missing Data Proportions in the 510k Cohort

No.	Variable	Unit	No. of missing values		
			Sepsis	SIRS	Total
			N = 176	N = 243	N = 419
1	SeptiScore	Range 1-15	0	0	0
2	PCT	ng/ml	27	45	72
3	Lactate	mmol/L	47	93	140
4	Glucose (Max)	mg/dL	8	21	29
5	Glucose (Min)	mg/dL	5	21	26
6	Respiratory Rate (Max)	breaths per minute	53	71	124
7	Respiratory Rate (Min)	breaths per minute	56	73	129
8	Mean Arterial Pressure (Max)	mmHg	41	54	95
9	Mean Arterial Pressure (Min)	mmHg	2	7	9
10	Temperature (Max)	ºC	19	34	53
11	Temperature (Min)	ºC	24	40	64
12	Heart Rate (Max)	beats per minute	0	0	0
13	Heart Rate (Min)	beats per minute	1	1	2
14	WBC (Max)	x 10^3 /uL	7	9	16
15	WBC (Min)	x 10^3 /uL	5	7	12
16	Platelets (Min)	x 10^3 /uL	52	70	122
17	Age	years	0	0	0
18	SOFA score	categorical variable	19	27	46
19	SOFA Cardiovascular component	categorical variable	22	29	51
20	SOFA Respiratory component	categorical variable	43	73	116
21	SOFA Liver component	categorical variable	30	63	93
22	SOFA Coagulation component	categorical variable	19	31	50
23	SOFA Renal component	categorical variable	20	36	56
24	SOFA CNS component	categorical variable	25	33	58
25	qSOFA	categorical variable	16	15	31
26	RR (max) >=22 (qSOFA)	categorical variable	53	71	124

No.	Variable	Unit	No. of missing values		
			Sepsis	SIRS	Total
			N = 176	N = 243	N = 419
27	SBP (min)<=100 (qSOFA)	categorical variable	96	123	219
28	Sex	categorical variable	0	0	0
29	African American or not	categorical variable	0	0	0
30	SIRS criteria	categorical variable	0	0	0
31	Bacterial pathogen	categorical variable	0	0	0
32	Viral pathogen	categorical variable	0	0	0
33	Fungal pathogen	categorical variable	0	0	0
34	Other culture	categorical variable	0	0	0
35	Urine (site of infection)	categorical variable	0	0	0
36	Abdominal (site of infection)	categorical variable	0	0	0
37	Blood (site of infection)	categorical variable	1	67	68
38	Central Nervous System (site of infection)	categorical variable	1	67	68
39	Other (site of infection)	categorical variable	0	0	0
40	Pulmonary (site of infection)	categorical variable	1	67	68
41	Blood culture	categorical variable	0	0	0
42	Urine culture	categorical variable	0	0	0

Supplementary Table S2. List of Variables and Missing Data Proportions in the Andalusian Cohort

No.	Variable	Unit	No. of missing values		
			**Sepsis**	**SIRS**	**Total**
			**N = 267**	**N = 86**	**N=353**
1	SeptiScore	Range 1-15	0	0	0
2	CRP	mg/L	3	1	4
3	PCT	ng/ml	4	4	8
4	Lactate	mmol/L	0	0	0
5	WBC (Leucocytes)	cells/µl	0	0	0
6	Age	years	0	0	0
7	Temperature	ºC	0	0	0
8	Heart Rate	beats per minute	0	0	0
9	Respiratory Rate	breaths per minute	0	0	0
10	Mean Arterial Pressure	mmHg	0	0	0
11	Diastolic Blood Pressure	mmHg	0	0	0
12	Systolic Blood Pressure	mmHg	0	0	0
13	Platelets	cells/µl	0	0	0
14	Creatinine	mg/dL	0	0	0
15	Neutrophils	% WBC	0	0	0
16	Lymphocytes	% WBC	0	0	0
17	Eosinophils	% WBC	0	0	0
18	Hemoglobin	g/dL	0	0	0
19	Urea	mg/dL	9	7	16
20	SOFA (min) at admission	categorical variable	0	0	0
21	APACHE-II score	categorical variable	0	0	0
22	Bacterial pathogen identified	categorical variable	0	0	0
23	Viral pathogen identified	categorical variable	0	0	0
24	Fungal pathogen identified	categorical variable	0	0	0
25	Sex	categorical variable	0	0	0
26	Blood Culture Positive	categorical variable	0	0	0
27	Other Culture Positive	categorical variable	0	0	0

No.	Variable	Unit	No. of missing values		
			Sepsis	SIRS	Total
			N = 267	N = 86	N=353
28	Urine Culture Positive	categorical variable	0	0	0
29	Abdominal (site of infection)	categorical variable	0	0	0
30	Pulmonary (site of infection)	categorical variable	0	0	0
31	Blood (site of infection)	categorical variable	0	0	0
32	Urinary (site of infection)	categorical variable	0	0	0
33	Central Nervous System (site of infection)	categorical variable	0	0	0
34	Other (site of infection)	categorical variable	0	0	0
35	SBP<=100 (qSOFA)	categorical variable	0	0	0
36	RR>=22 (qSOFA)	categorical variable	0	0	0
37	qSOFA (partial, no GCS)	categorical variable	0	0	0
38	SIRS criteria	categorical variable	0	0	0

Supplementary Table S3. Overlap of Variables Between Analyses of 510k and Andalusian Cohorts

1. Variables in common

ID #	510k analysis	Andalusia analysis
	Variable	Variable
1	SeptiScore	SeptiScore
2	PCT	PCT
3	Lactate	Lactate
6	Respiratory Rate (Max)	Respiratory Rate
7	Respiratory Rate (Min)	
8	Mean Arterial Pressure (Max)	Mean Arterial Pressure
9	Mean Arterial Pressure (Min)	
10	Temperature (Max)	Temperature
11	Temperature (Min)	
12	Heart Rate (Max)	Heart Rate
13	Heart Rate (Min)	
14	WBC (Max)	WBC (Leucocytes)
15	WBC (Min)	
16	Platelets (Min)	Platelets
17	Age	Age
18	SOFA score	SOFA (min) at admission
25	qSOFA	qSOFA (partial, no GCS)
26	RR (max) >=22 (qSOFA)	RR>=22 (qSOFA)
27	SBP (min)<=100 (qSOFA)	SBP<=100 (qSOFA)
28	Sex	Sex
30	SIRS criteria	SIRS criteria
31	Bacterial pathogen	Bacterial pathogen
32	Viral pathogen	Viral pathogen
33	Fungal pathogen	Fungal pathogen
34	Other culture positive	Other culture positive
35	Urinary (site of infection)	Urinary (site of infection)
36	Abdominal (site of infection)	Abdominal (site of infection)
37	Blood (site of infection)	Blood (site of infection)
38	Central Nervous System (site of infection)	Central Nervous System (site of infection)
39	Other (site of infection)	Other (site of infection)
40	Pulmonary (site of infection)	Pulmonary (site of infection)
41	Blood culture positive	Blood culture positive
42	Urine culture positive	Urine culture positive

2. Variables in the Andalusian Analysis, but not in the 510k analysis

No.	Variable
1	CRP
2	Diastolic Blood Pressure
3	Systolic Blood Pressure
4	Creatinine
5	Neutrophils
6	Lymphocytes
7	Eosinophils
8	Hemoglobin
9	Urea
10	APACHE-II score

3. Variable in the 510k Analysis, but not in the Andalusian Analysis

No.	Variable
1	Glucose (Max)
2	Glucose (Min)
3	SOFA Cardiovascular component
4	SOFA Respiratory component
5	SOFA Liver component
6	SOFA Coagulation component
7	SOFA Renal component
8	SOFA CNS component
9	African American or not

Supplementary Figure S1 (A-F). Greedy search plots showing performance (AUC) of various clinical variables available at time T1 (presentation), for various initially suspected sites of infection. Data from 510k cohort. A: Abdominal, B: Urinary, C: Blood, D: CNS, E: Other, F: Site of Infection not identified.

A: Abdominal Initial Impression of Site of Infection	B: Urinary Initial Impression of Site of Infection
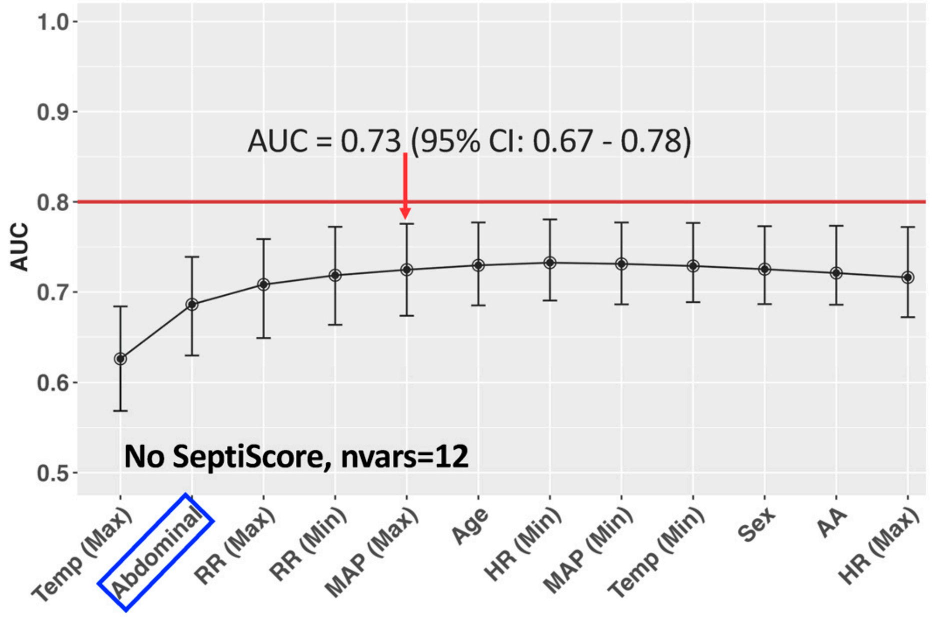	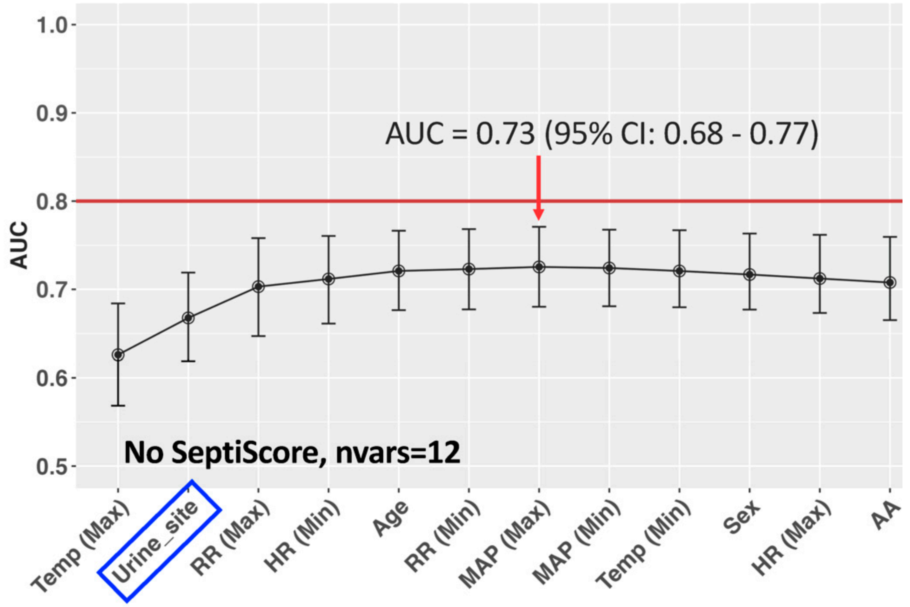

C: Blood Initial Impression of Site of Infection	D: CNS Initial Impression of Site of Infection
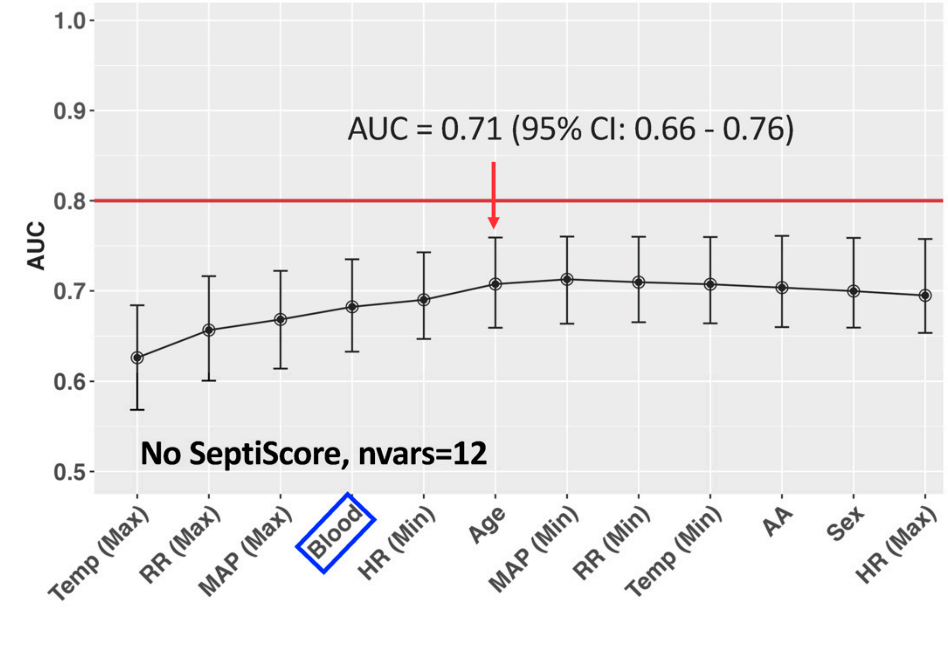	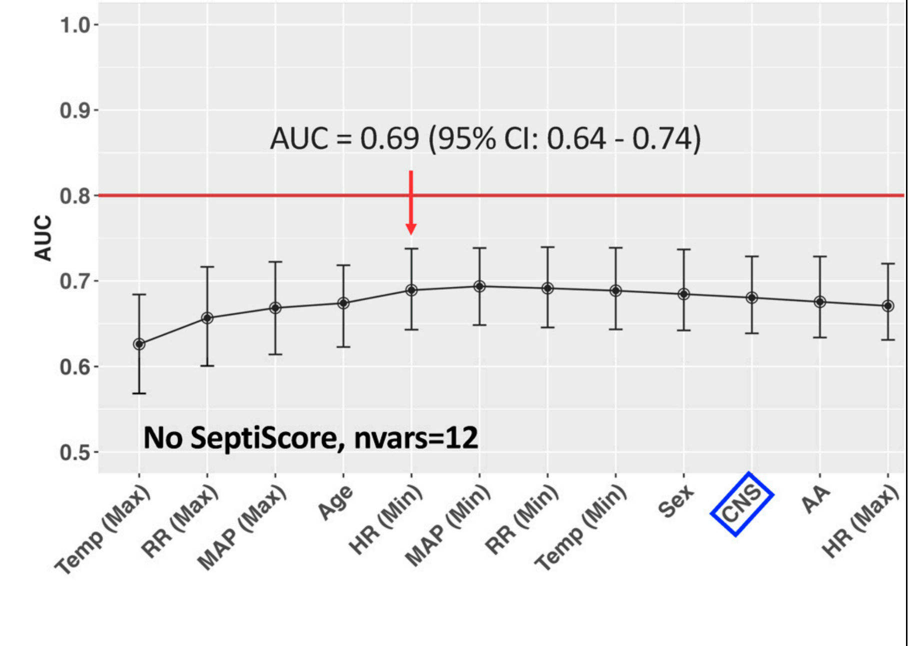

E: Other Initial Impression of Site of Infection	F: No Initial Site of Infection
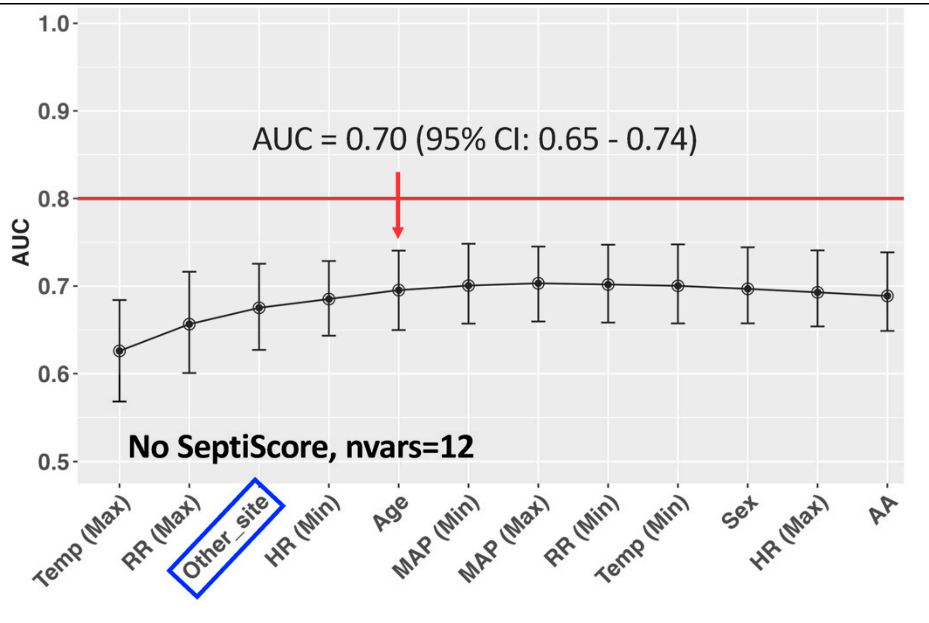	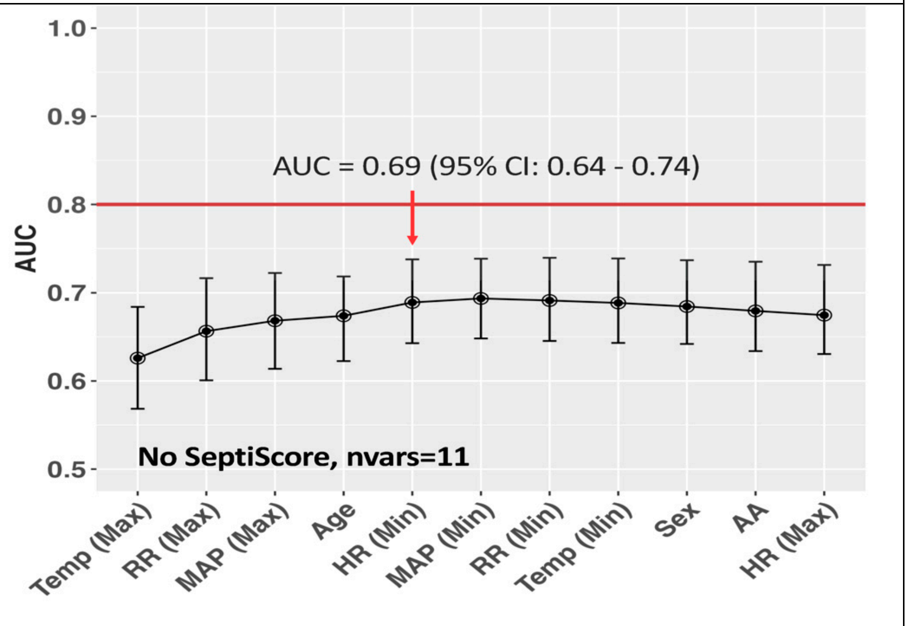

Supplementary Figure S2 (A-F). Greedy search plots showing performance (AUC) of various clinical variables available at Time T2 (in the first 1-3 hours) for various sites of infection without (left panel) and with SeptiCyte RAPID (right panel), including A: Abdominal, B: Urinary, C: Blood, D: CNS and E: Other. F: No site of infection identified/ documented at initial clinical impression. Data from 510k cohort.

A: Abdominal

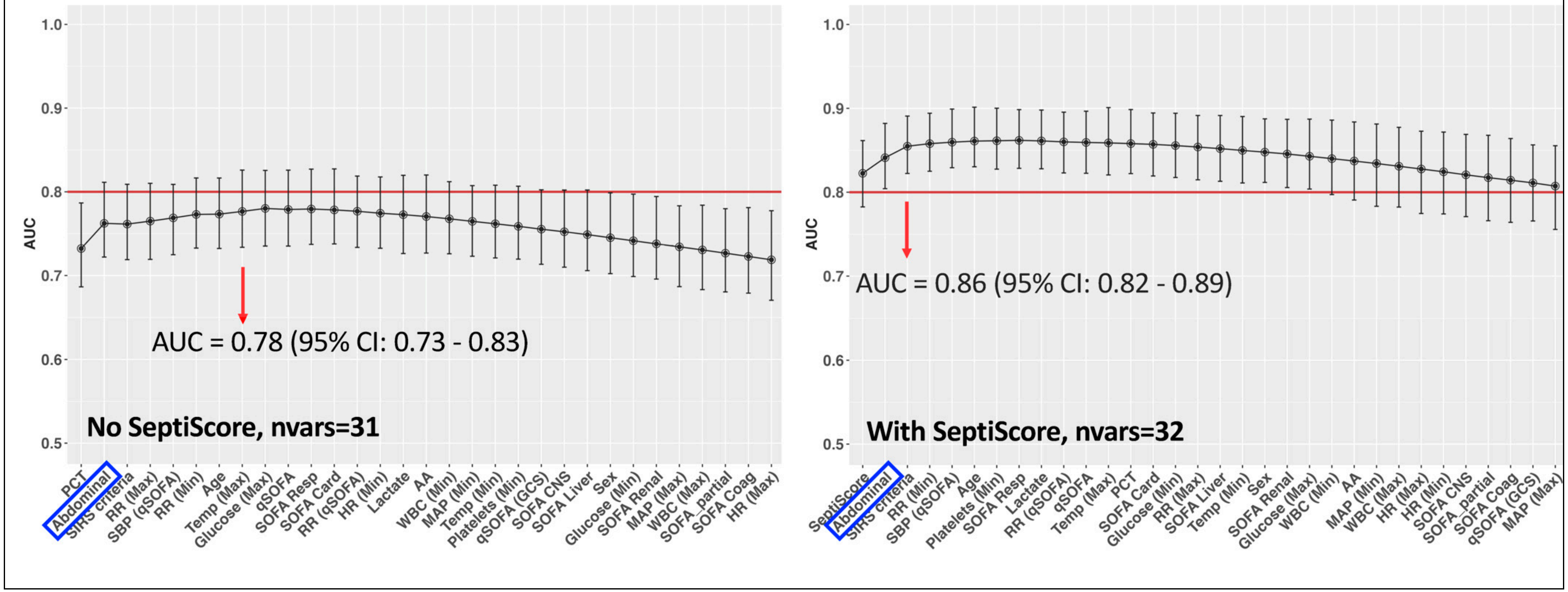

B: Urinary

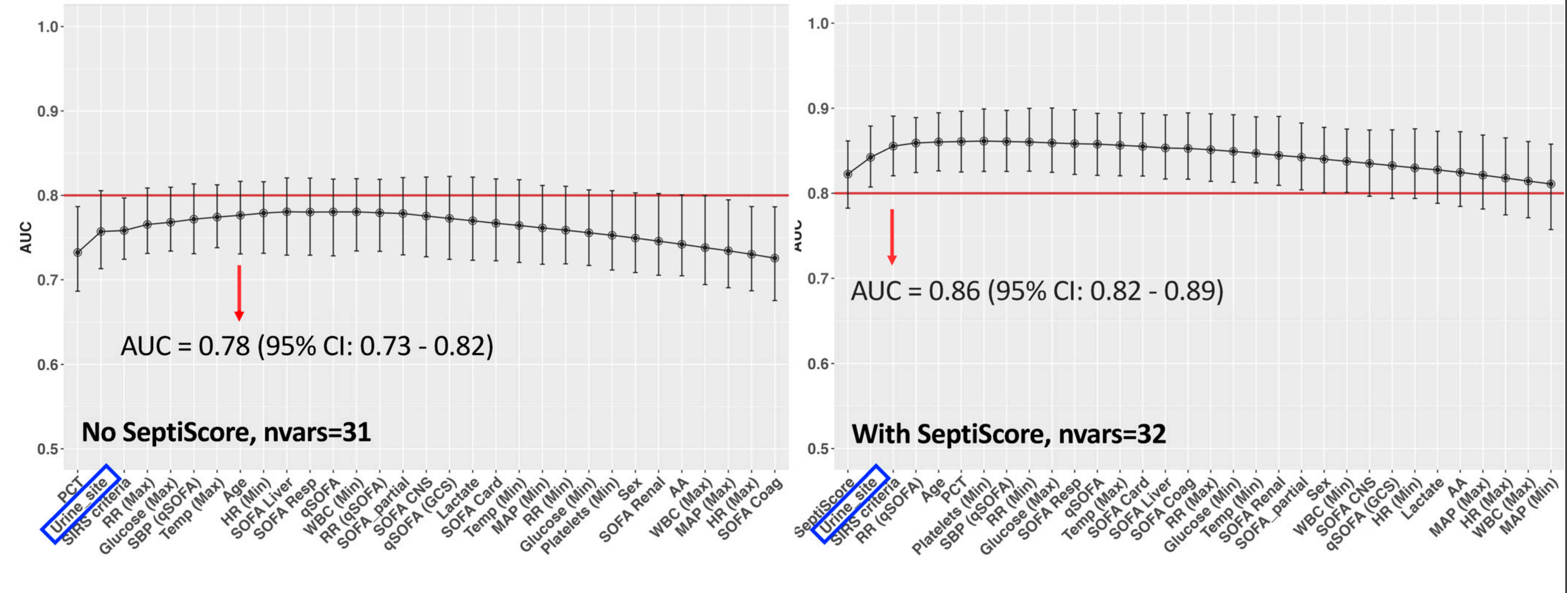
AUC = 0.78 (95% CI: 0.73 - 0.82)
No SeptiScore, nvars=31
AUC
PCT
Urine_site
SIRS criteria
RR (Max)
Glucose (Max)
SBP (qSOFA)
Temp (Max)
Age
HR (Min)
SOFA Liver
SOFA Resp
qSOFA
WBC (Min)
RR (qSOFA)
SOFA_partial
SOFA CNS
qSOFA (GCS)
Lactate
SOFA Card
Temp (Min)
MAP (Min)
RR (Min)
Glucose (Min)
Platelets (Min)
Sex
SOFA Renal
AA
WBC (Max)
MAP (Max)
HR (Max)
SOFA Coag
AUC = 0.86 (95% CI: 0.82 - 0.89)
With SeptiScore, nvars=32
SeptiScore
Urine_site
SIRS criteria
RR (qSOFA)
Age
PCT
Platelets (Min)
SBP (qSOFA)
RR (Min)
Glucose (Max)
SOFA Resp
qSOFA
Temp (Max)
SOFA Card
SOFA Liver
SOFA Coag
RR (Max)
Glucose (Min)
Temp (Min)
SOFA Renal
SOFA_partial
Sex
WBC (Min)
SOFA CNS
qSOFA (GCS)
HR (Min)
Lactate
AA
MAP (Max)
HR (Max)
WBC (Max)
MAP (Min)

C: Blood (Bacteremia)

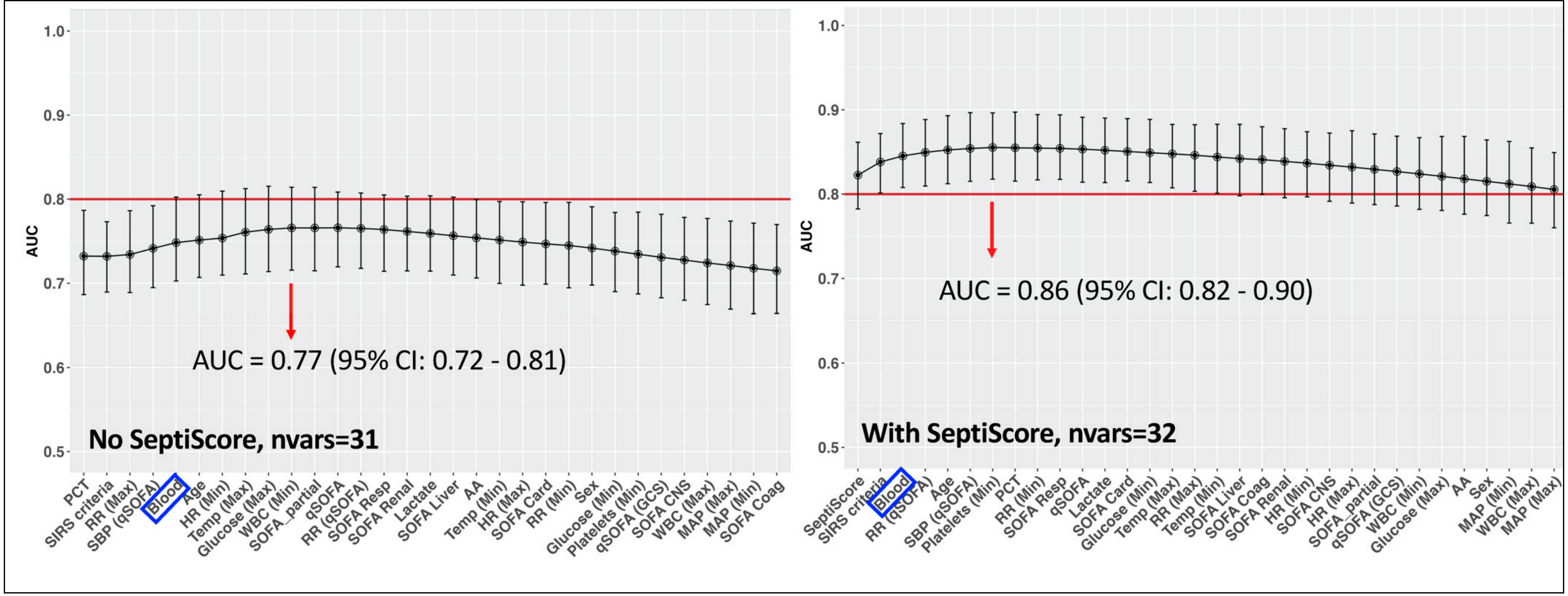

D: CNS

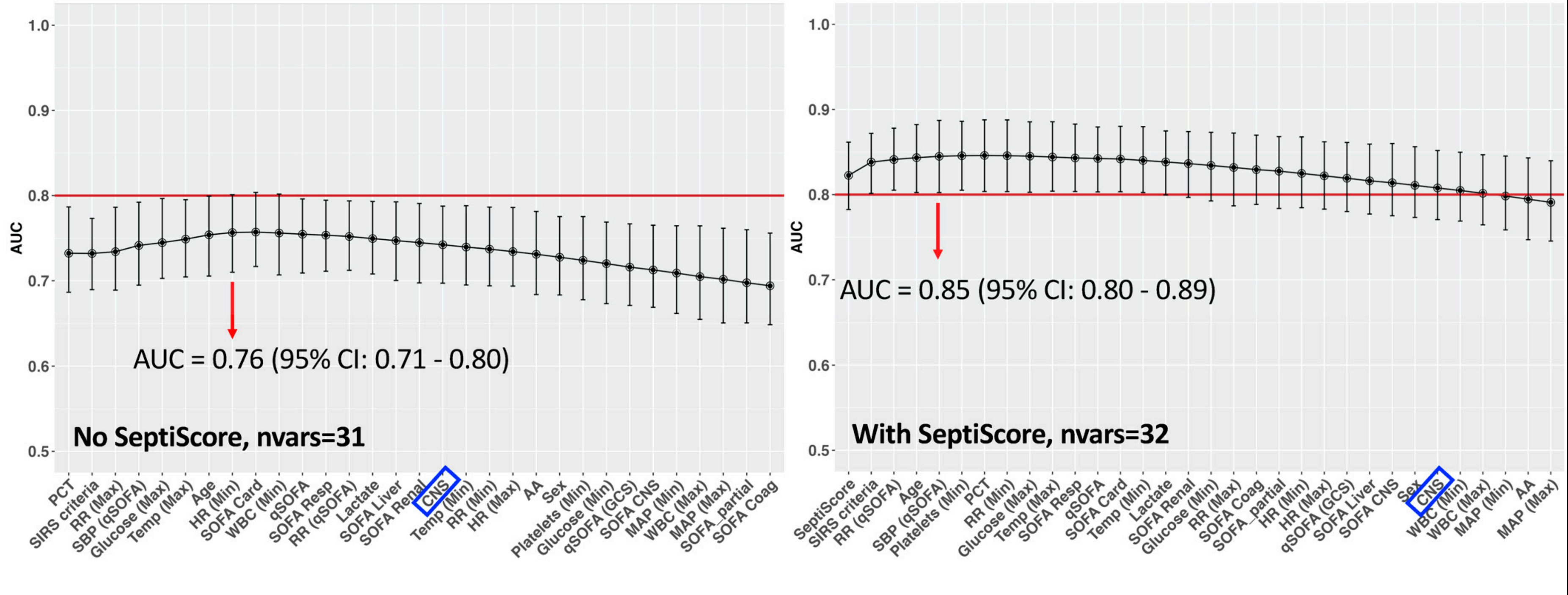

AUC
1.0
0.9
0.8
0.7
0.6
0.5
AUC = 0.76 (95% CI: 0.71 - 0.80)
No SeptiScore, nvars=31
PCT
SIRS criteria
RR (Max)
SBP (qSOFA)
Glucose (Max)
Temp (Max)
Age
HR (Min)
SOFA Card
WBC (Min)
qSOFA
SOFA Resp
RR (qSOFA)
Lactate
SOFA Liver
SOFA Renal
CNS
Temp (Min)
RR (Min)
HR (Max)
AA
Sex
Platelets (Min)
Glucose (Min)
qSOFA (GCS)
SOFA CNS
MAP (Min)
WBC (Max)
MAP (Max)
SOFA_partial
SOFA Coag
AUC = 0.85 (95% CI: 0.80 - 0.89)
With SeptiScore, nvars=32
SeptiScore
SIRS criteria
RR (qSOFA)
Age
SBP (qSOFA)
Platelets (Min)
PCT
RR (Min)
Glucose (Max)
Temp (Max)
SOFA Resp
qSOFA
SOFA Card
Temp (Min)
Lactate
SOFA Renal
Glucose (Min)
RR (Max)
SOFA Coag
SOFA_partial
HR (Min)
HR (Max)
qSOFA (GCS)
SOFA Liver
SOFA CNS
Sex
CNS
WBC (Min)
WBC (Max)
MAP (Min)
AA
MAP (Max)

E: Other Site of Infection

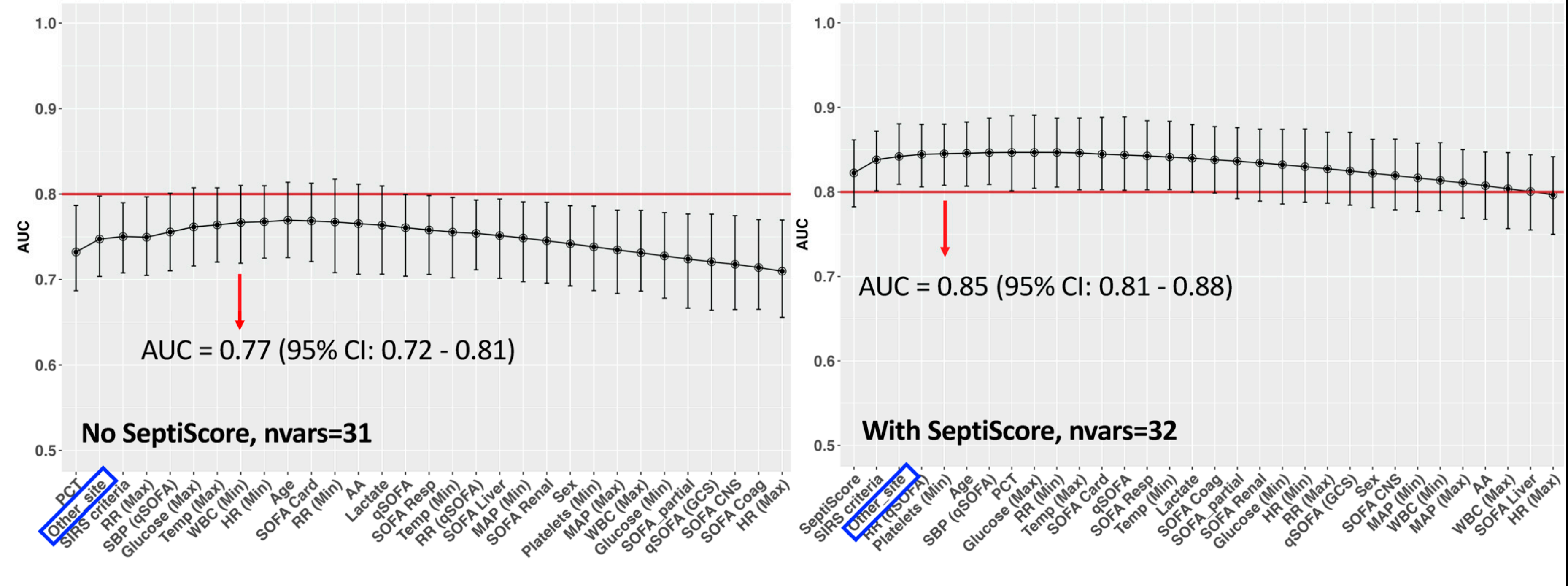

F: No Site of Infection identified/ documented at initial clinical impression

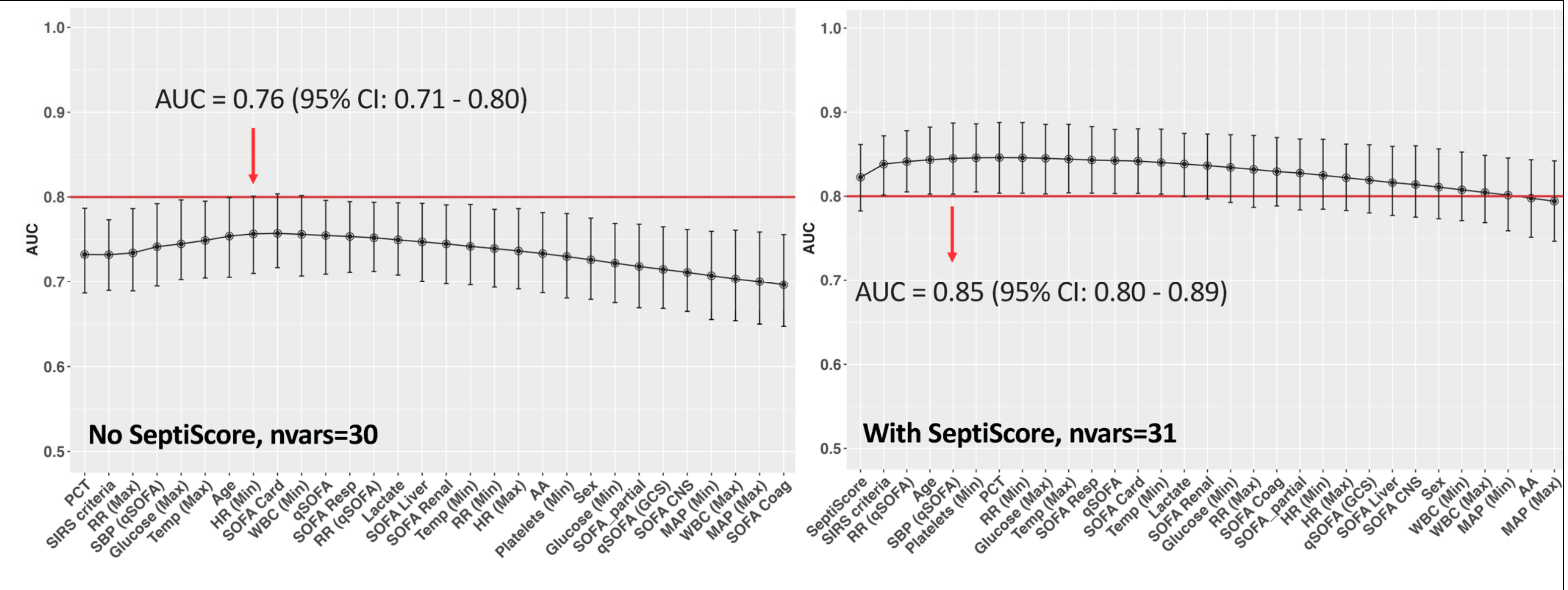

Supplementary Figure S3 (A-F). Greedy search plots showing performance (AUC) of various clinical variables available at Time T3 (within 1-3 days) for various sites of infection without (top panel) and with SeptiCyte RAPID (bottom panel), including A: Abdominal, B: Urinary, C: Blood, D: CNS, E: Other and F: No initial impression of site of infection. Data from 510k cohort.

A: Abdominal

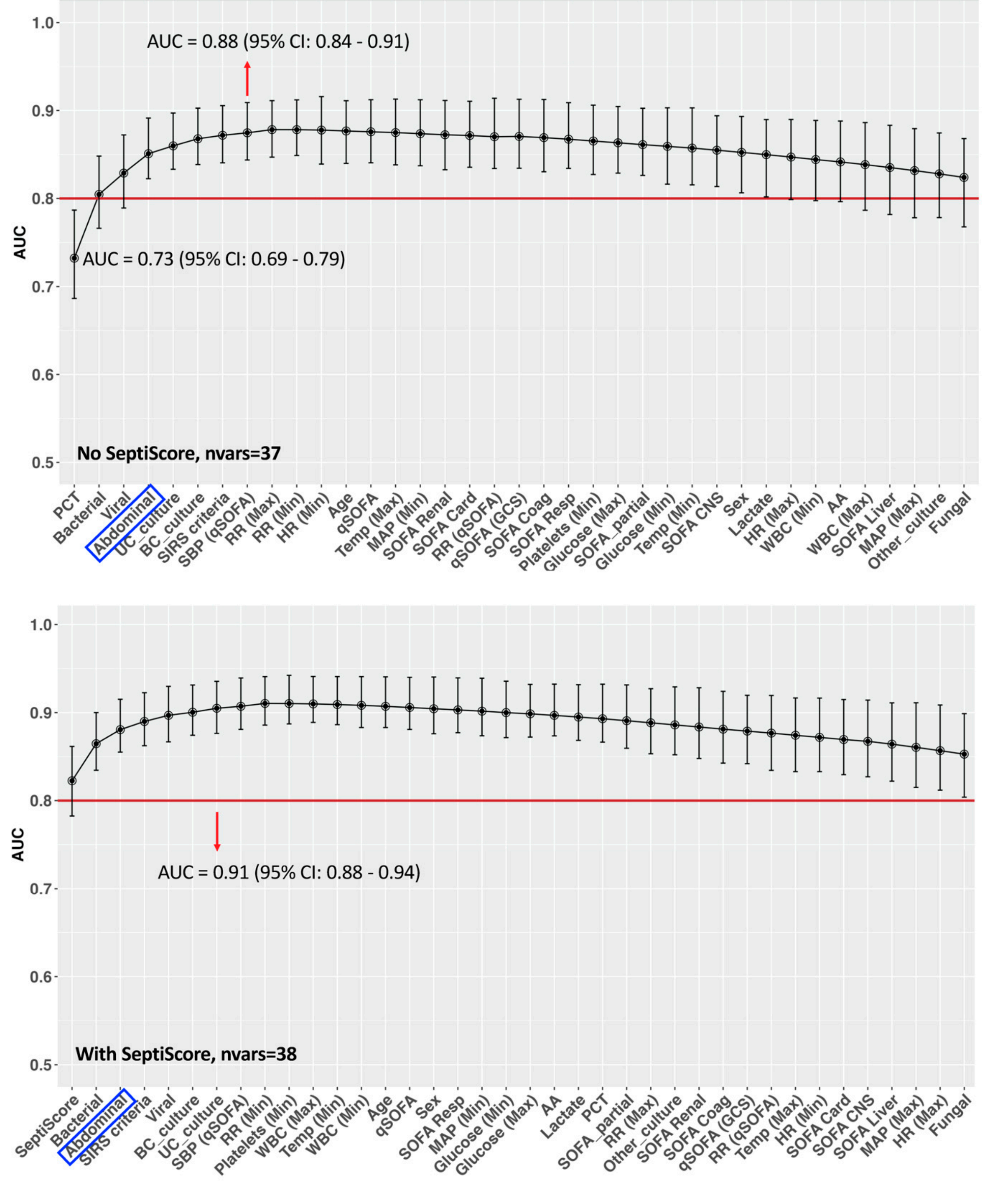

B: Urinary

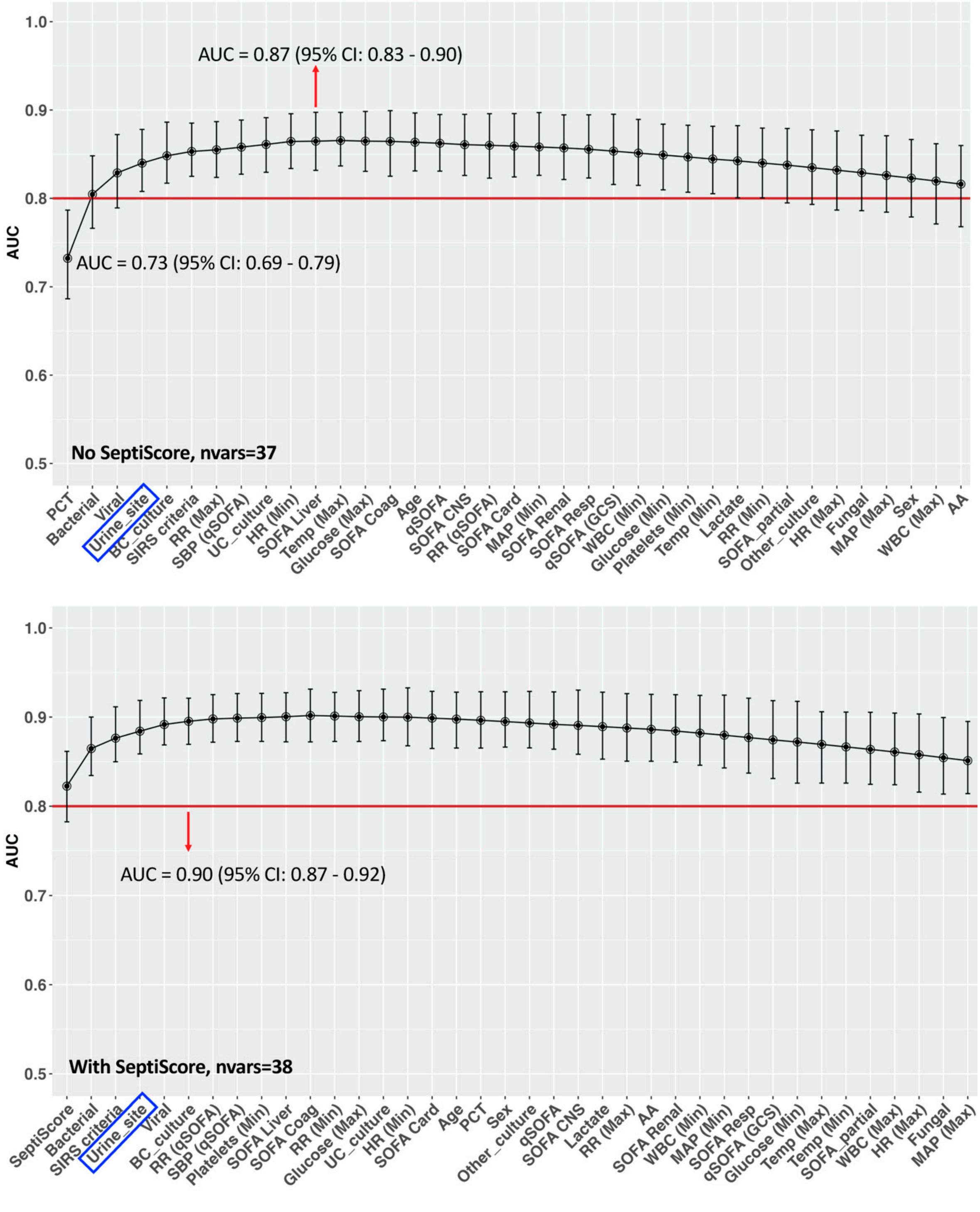

AUC = 0.87 (95% CI: 0.83 - 0.90)
AUC = 0.73 (95% CI: 0.69 - 0.79)
No SeptiScore, nvars=37
AUC
PCT
Bacterial
Viral
Urine_site
BC_culture
SIRS criteria
RR (Max)
SBP (qSOFA)
UC_culture
HR (Min)
SOFA Liver
Temp (Max)
Glucose (Max)
SOFA Coag
Age
qSOFA
SOFA CNS
RR (qSOFA)
SOFA Card
MAP (Min)
SOFA Renal
SOFA Resp
qSOFA (GCS)
WBC (Min)
Glucose (Min)
Platelets (Min)
Temp (Min)
Lactate
RR (Min)
SOFA_partial
Other_culture
HR (Max)
Fungal
MAP (Max)
Sex
WBC (Max)
AA
AUC = 0.90 (95% CI: 0.87 - 0.92)
With SeptiScore, nvars=38
AUC
SeptiScore
Bacterial
SIRS criteria
Urine_site
Viral
BC_culture
RR (qSOFA)
SBP (qSOFA)
Platelets (Min)
SOFA Liver
SOFA Coag
RR (Min)
Glucose (Max)
UC_culture
HR (Min)
SOFA Card
Age
PCT
Sex
Other_culture
qSOFA
SOFA CNS
Lactate
RR (Max)
AA
SOFA Renal
WBC (Min)
MAP (Min)
SOFA Resp
qSOFA (GCS)
Glucose (Min)
Temp (Max)
Temp (Min)
SOFA_partial
WBC (Max)
HR (Max)
Fungal
MAP (Max)

C: Blood (Bacteremia)

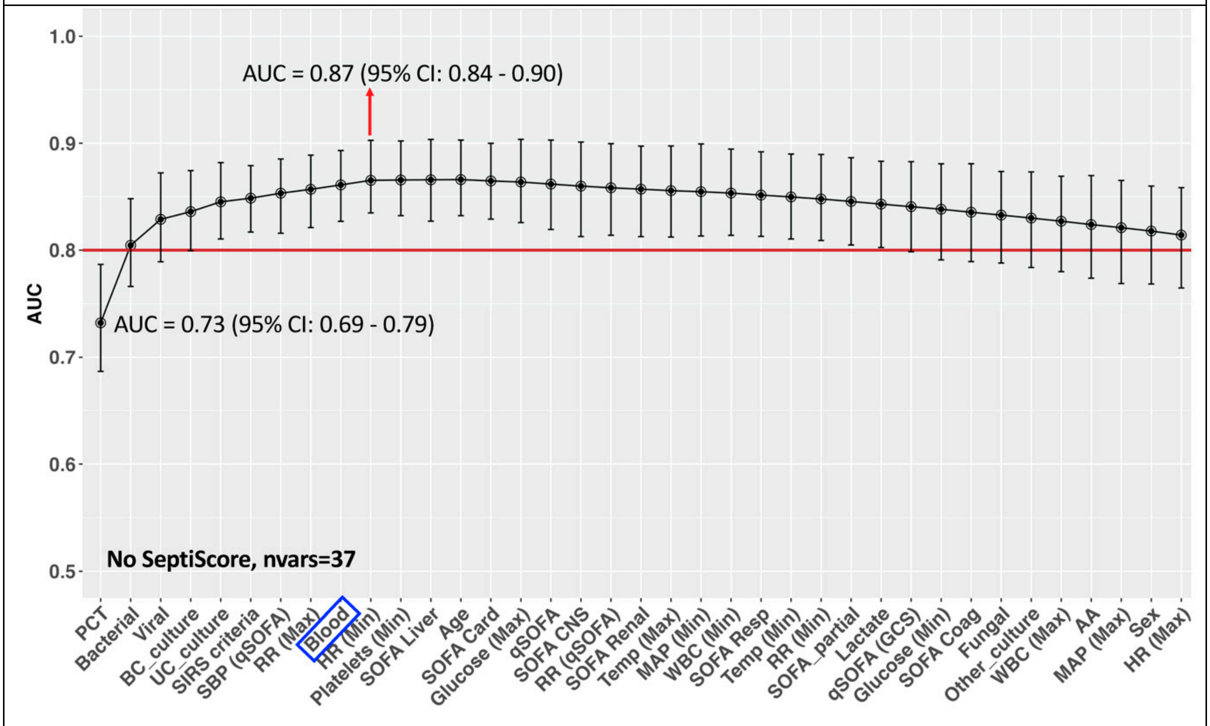

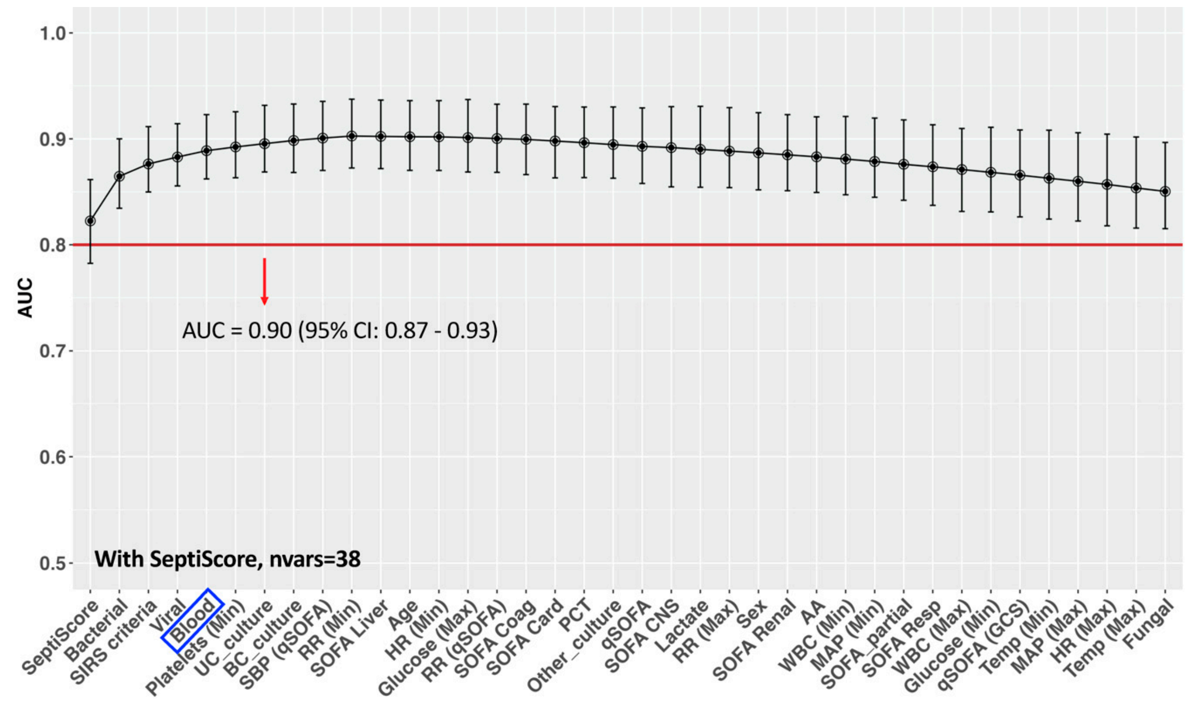

D: CNS

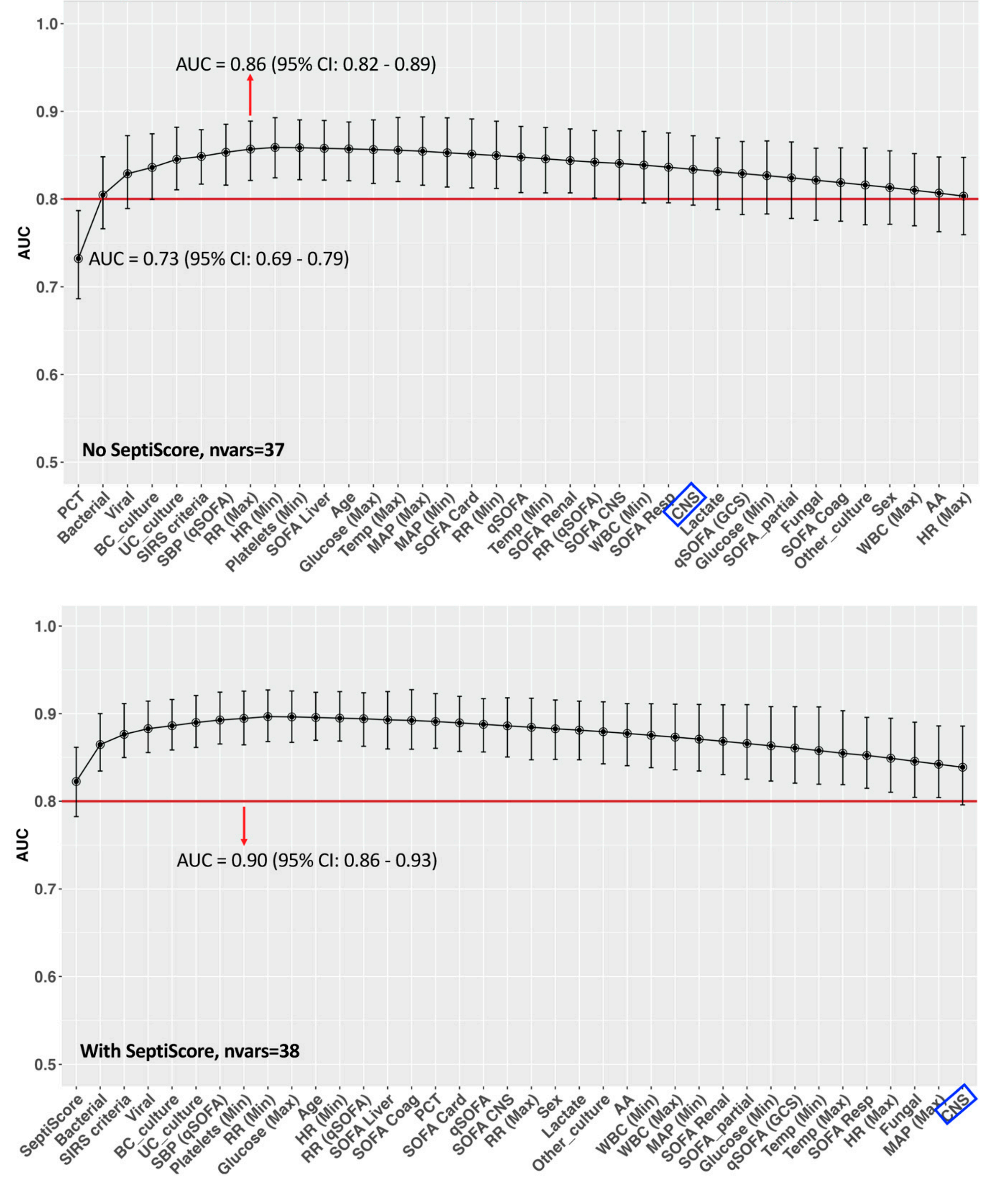

AUC
1.0
0.9
0.8
0.7
0.6
0.5
AUC = 0.86 (95% CI: 0.82 - 0.89)
AUC = 0.73 (95% CI: 0.69 - 0.79)
No SeptiScore, nvars=37
PCT
Bacterial
Viral
BC_culture
UC_culture
SIRS criteria
SBP (qSOFA)
RR (Max)
HR (Min)
Platelets (Min)
SOFA Liver
Age
Glucose (Max)
Temp (Max)
MAP (Max)
MAP (Min)
SOFA Card
RR (Min)
qSOFA
Temp (Min)
SOFA Renal
RR (qSOFA)
SOFA CNS
WBC (Min)
SOFA Resp
CNS
Lactate
qSOFA (GCS)
Glucose (Min)
SOFA_partial
Fungal
SOFA Coag
Other_culture
Sex
WBC (Max)
AA
HR (Max)
AUC = 0.90 (95% CI: 0.86 - 0.93)
With SeptiScore, nvars=38
SeptiScore
Bacterial
SIRS criteria
Viral
BC_culture
UC_culture
SBP (qSOFA)
Platelets (Min)
RR (Min)
Glucose (Max)
Age
HR (Min)
RR (qSOFA)
SOFA Liver
SOFA Coag
PCT
SOFA Card
qSOFA
SOFA CNS
RR (Max)
Sex
Lactate
Other_culture
AA
WBC (Min)
WBC (Max)
MAP (Min)
SOFA Renal
SOFA_partial
Glucose (Min)
qSOFA (GCS)
Temp (Min)
Temp (Max)
SOFA Resp
HR (Max)
Fungal
MAP (Max)
CNS

E: Other Site of Infection

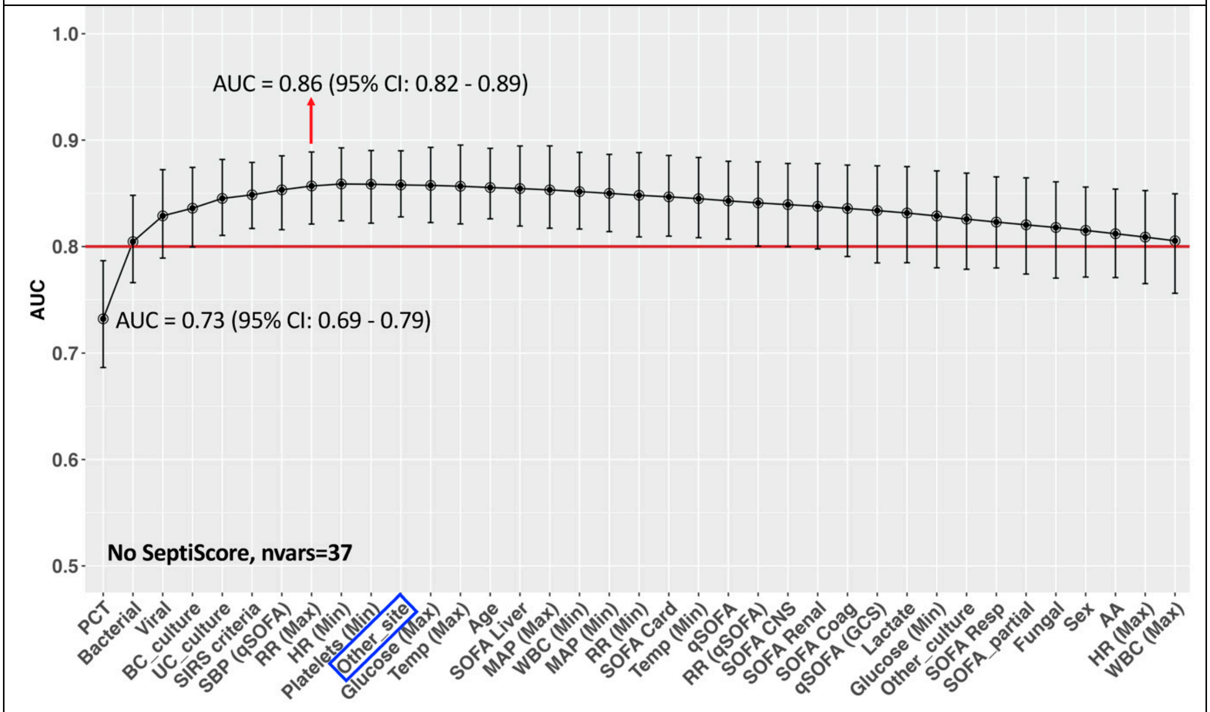

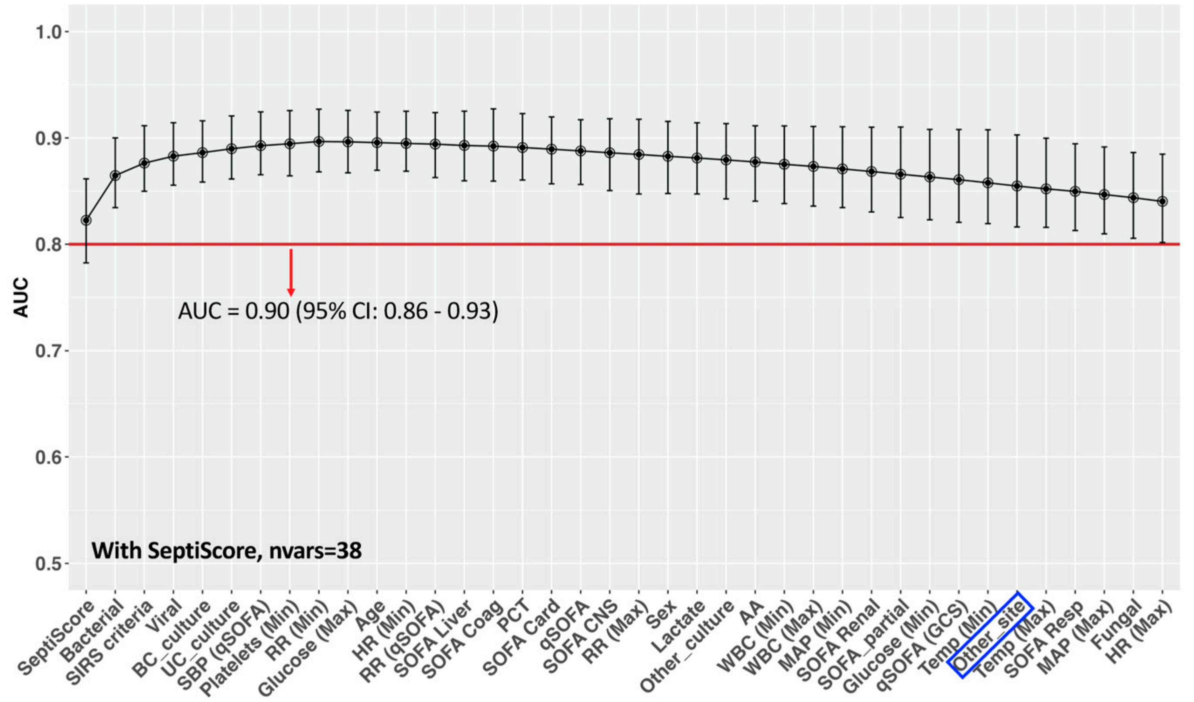

F: No Site of Infection identified/ documented at Initial Impression

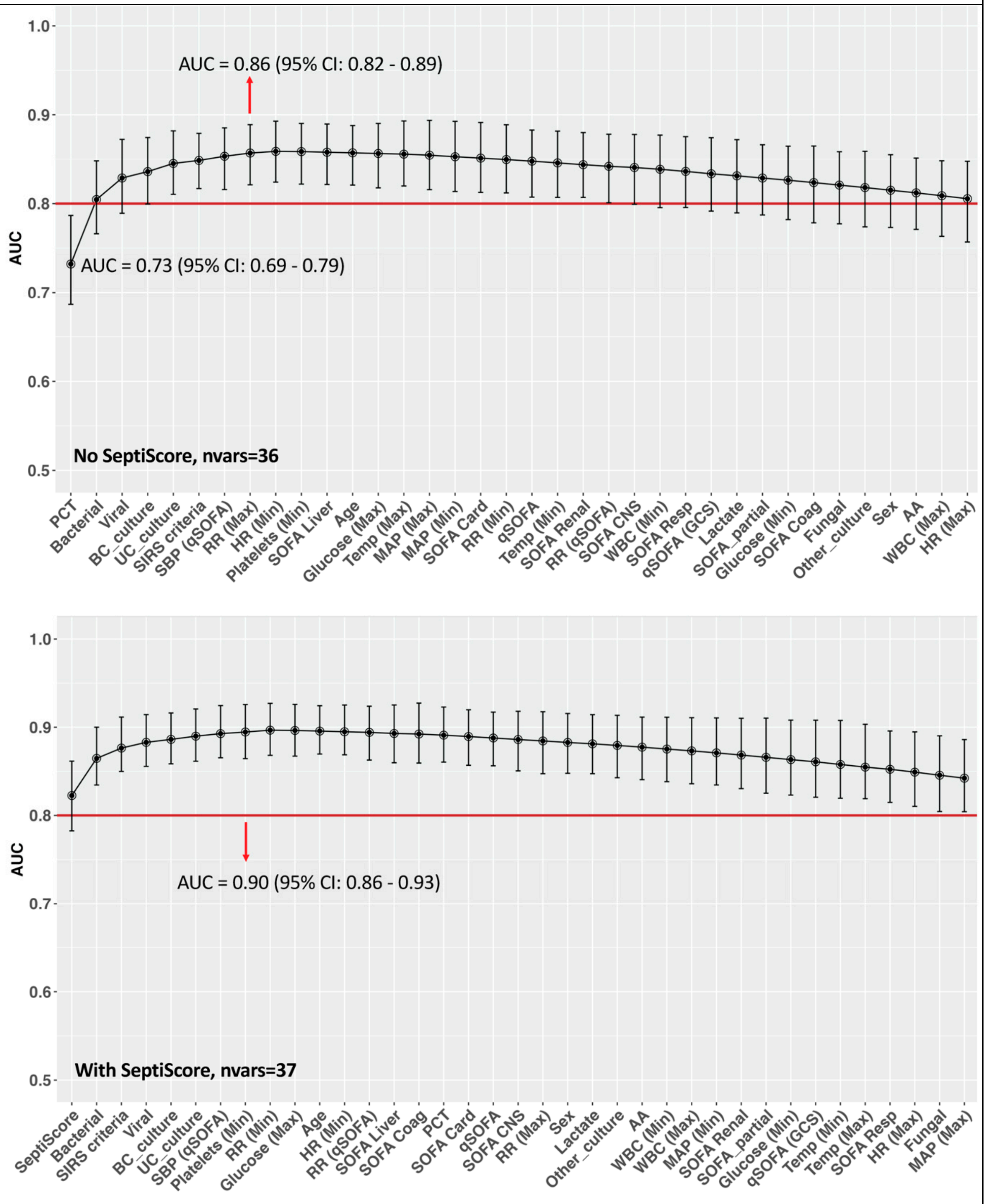

Supplementary Table S4. Greedy Search Results for the Andalusian Cohort, at Time T1 (Presentation).

Source of infection	Starting Mean AUC (95% CI)	Maximum possible Mean AUC (95% CI)	Variable 1	Variable 2	Variable 3	Variable 4	Variable 5	Variables sorted by order of importance	No. of variables needed to reach max performance
No site of infection	0.648 (0.585 - 0.731)	0.671 (0.616 - 0.738)	DBP	HR	Sex			DBP, HR, Sex	3
Pulmonary		0.671 (0.616 - 0.738)	DBP	HR	Sex			DBP, HR, Sex	3
Abdominal		0.671 (0.616 - 0.738)	DBP	HR	Sex			DBP, HR, Sex	3
Bacteremia		0.68 (0.63 - 0.741)	DBP	HR	Sex	Blood		DBP, HR, Sex, Blood	4
Urine_site		0.671 (0.616 - 0.738)	DBP	HR	Sex			DBP, HR, Sex	3
CNS		0.671 (0.616 - 0.738)	DBP	HR	Sex			DBP, HR, Sex	3
Other site		0.68 (0.625 - 0.742)	DBP	HR	Other site	Sex		DBP, HR, Other site, Sex	4

Supplementary Table S5. Greedy Search Results for the Andalusian Cohort at Time T2 (1-3 Hours Post-Presentation) Without SeptiCyte RAPID.

Source of infection	Starting Mean AUC (95% CI)	Maximum possible Mean AUC (95% CI)	Variable 1	Variable 2	Variable 3	Variable 4	Variable 5	Variables sorted by order of importance	No. of variables needed to reach max performance
No site of infection	0.779 (0.727 - 0.836)	0.799 (0.75 - 0.848)	PCT	CRP	DBP	Creatinine		PCT, CRP, DBP, Creatinine	4
Pulmonary		0.799 (0.75 - 0.848)	PCT	CRP	DBP	Creatinine		PCT, CRP, DBP, Creatinine	4
Abdominal		0.799 (0.75 - 0.848)	PCT	CRP	DBP	Creatinine		PCT, CRP, DBP, Creatinine	4
Bacteremia		0.799 (0.75 - 0.848)	PCT	CRP	DBP	Creatinine		PCT, CRP, DBP, Creatinine	4
Urine		0.799 (0.75 - 0.848)	PCT	CRP	DBP	Creatinine		PCT, CRP, DBP, Creatinine	4
CNS		0.799 (0.75 - 0.848)	PCT	CRP	DBP	Creatinine		PCT, CRP, DBP, Creatinine	4
Other site		0.808 (0.757 - 0.859)	PCT	Other site	CRP	DBP	Creatinine	PCT, Other site, CRP, DBP, Creatinine, Platelets, Eosinophils, Sex, HR, RR	10

Supplementary Table S6. Greedy Search Results for the Andalusian Cohort at Time T2 (1-3 Hours Post-Presentation) With SeptiCyte RAPID.

Source of infection	Starting Mean AUC (95% CI)	Maximum possible Mean AUC (95% CI)	Variable 1	Variable 2	Variable 3	Variable 4	Variable 5	Variables sorted by order of importance	No. of variables needed to reach max performance
No site of infection	0.839 (0.786 - 0.902)	0.867 (0.825 - 0.913)	SeptiScore	DBP	Age	Creatinine		SeptiScore, DBP, Age, Creatinine	4
Pulmonary		0.867 (0.825 - 0.913)	SeptiScore	DBP	Age	Creatinine		SeptiScore, DBP, Age, Creatinine	4
Abdominal		0.867 (0.825 - 0.913)	SeptiScore	DBP	Age	Creatinine		SeptiScore, DBP, Age, Creatinine	4
Bacteremia		0.867 (0.825 - 0.913)	SeptiScore	DBP	Age	Creatinine		SeptiScore, DBP, Age, Creatinine	4
Urine		0.867 (0.825 - 0.913)	SeptiScore	DBP	Age	Creatinine		SeptiScore, DBP, Age, Creatinine	4
CNS		0.867 (0.825 - 0.913)	SeptiScore	DBP	Age	Creatinine		SeptiScore, DBP, Age, Creatinine	4
Other site		0.867 (0.825 - 0.913)	SeptiScore	DBP	Age	Creatinine		SeptiScore, DBP, Age, Creatinine	4

Supplementary Table S7. Greedy Search Results for the Andalusian Cohort at Time T3 (1-3 Days Post-Presentation) Without SeptiCyte RAPID.

Source of infection	Starting Mean AUC (95% CI)	Maximum possible Mean AUC (95% CI)	Variable 1	Variable 2	Variable 3	Variable 4	Variable 5	Variables sorted by order of importance	No. of variables needed to reach max performance
No site of infection	0.784 (0.76 - 0.812)	0.895 (0.864 - 0.924)	Bacterial infection	CRP	DBP	Fungal infection		Bacterial infection, CRP, DBP, Fungal infection	4
Pulmonary		0.895 (0.864 - 0.924)	Bacterial infection	CRP	DBP	Fungal infection		Bacterial infection, CRP, DBP, Fungal infection	4
Abdominal		0.895 (0.864 - 0.924)	Bacterial infection	CRP	DBP	Fungal infection		Bacterial infection, CRP, DBP, Fungal infection	4
Bacteremia		0.895 (0.864 - 0.924)	Bacterial infection	CRP	DBP	Fungal infection		Bacterial infection, CRP, DBP, Fungal infection	4
Urine		0.895 (0.864 - 0.924)	Bacterial infection	CRP	DBP	Fungal infection		Bacterial infection, CRP, DBP, Fungal infection	4
CNS		0.895 (0.864 - 0.924)	Bacterial infection	CRP	DBP	Fungal infection		Bacterial infection, CRP, DBP, Fungal infection	4
Other site		0.906 (0.877 - 0.931)	Bacterial infection	CRP	DBP	Fungal infection	Other site	Bacterial infection, CRP, DBP, Fungal infection, Other site, BC Positive, Creatinine	7

Supplementary Table S8. Greedy Search Results for the Andalusian Cohort at Time T3 (1-3 Days Post-Presentation) With SeptiCyte RAPID.

Source of infection	Starting Mean AUC (95% CI)	Maximum possible Mean AUC (95% CI)	Variable 1	Variable 2	Variable 3	Variable 4	Variable 5	Variables sorted by order of importance	No. of variables needed to reach max performance
No site of infection	0.839 (0.786 - 0.902)	0.927 (0.9 - 0.949)	SeptiScore	Bacterial infection	DBP	Blood culture positive		SeptiScore, Bacterial infection, DBP, Blood Culture Positive	4
Pulmonary		0.927 (0.9 - 0.949)	SeptiScore	Bacterial infection	DBP	Blood culture positive		SeptiScore, Bacterial infection, DBP, Blood Culture Positive	4
Abdominal		0.927 (0.9 - 0.949)	SeptiScore	Bacterial infection	DBP	Blood culture positive		SeptiScore, Bacterial infection, DBP, Blood Culture Positive	4
Bacteremia		0.927 (0.9 - 0.949)	SeptiScore	Bacterial infection	DBP	Blood culture positive		SeptiScore, Bacterial infection, DBP, Blood Culture Positive	4
Urine		0.927 (0.9 - 0.949)	SeptiScore	Bacterial infection	DBP	Blood culture positive		SeptiScore, Bacterial infection, DBP, Blood Culture Positive	4
CNS		0.927 (0.9 - 0.949)	SeptiScore	Bacterial infection	DBP	Blood culture positive		SeptiScore, Bacterial infection, DBP, Blood Culture Positive	4

Source of infection	Starting Mean AUC (95% CI)	Maximum possible Mean AUC (95% CI)	Variable 1	Variable 2	Variable 3	Variable 4	Variable 5	Variables sorted by order of importance	No. of variables needed to reach max performance
Other site		0.936 (0.911 - 0.956)	SeptiScore	Bacterial infection	DBP	Other site	Fungal infection	SeptiScore, Bacterial infection, DBP, Other site, Fungal infection	5

Supplementary Figure S4 (A-B). Investigation of potential incorporation bias, by comparative ROC curve analysis. **(A)** Baseline ROC curve for seven variables (AUC 0.85, 95% CI 0.80-0.89), and ROC curves generated by removing variables individually. The seven variables were SeptiCyte RAPID, PCT, Temperature (max), RR (max), MAP (max), age, and HR (min). The orange curve (AUC 0.75, 95% CI 0.70-0.79) results from removing SeptiScore from the logistic equation. Removal of other variables individually have only small detrimental effects. **(B)** ROC curve for SeptiScore + PCT (not subject to incorporation bias), with AUC 0.83 (95% CI 0.78-0.87; purple curve). Removal of PCT reduces the AUC to 0.82 (95% CI 0.78-0.86; green curve), while removal of SeptiScore reduces the AUC to 0.73 (95% CI 0.68-0.78; orange curve).

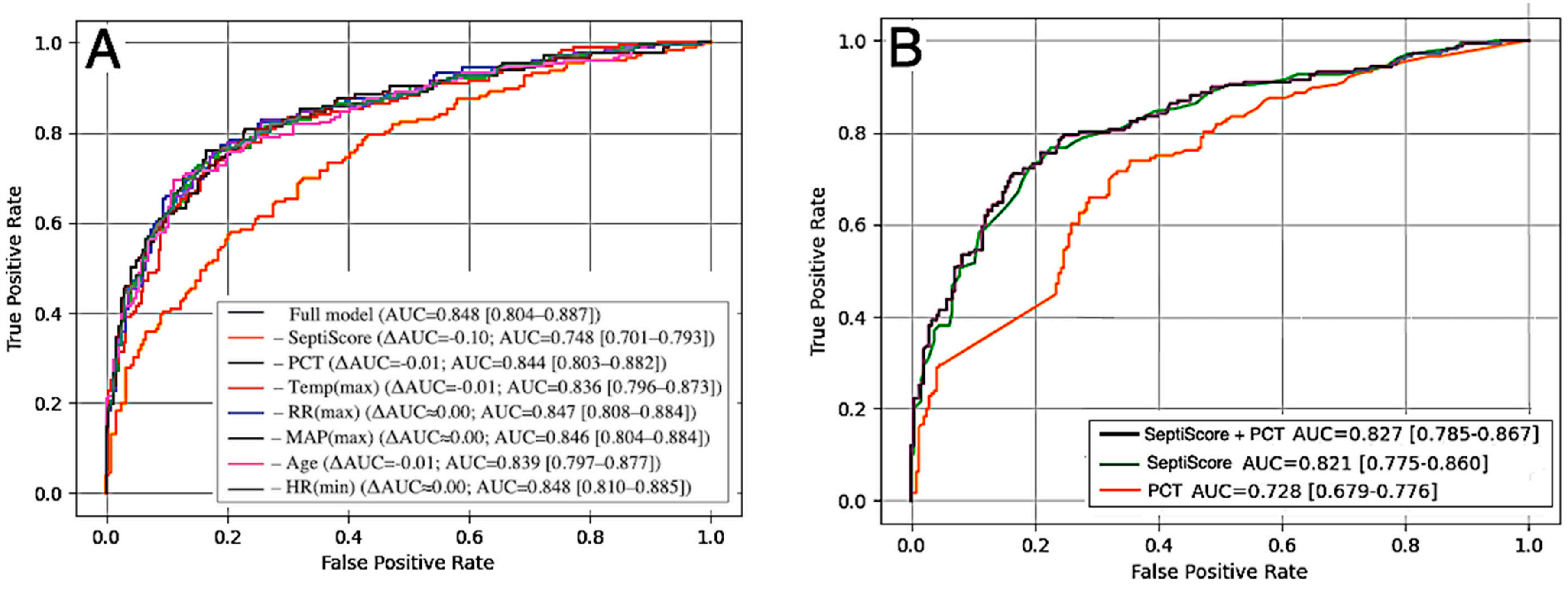

Supplementary Table S9. Relative magnitude of contributions of logistic regression input variables, arranged in decreasing order of impact upon the output AUC. Change in AUC is measured relative to the baseline logistic regression fit using all seven variables (AUC 0.85; 95% CI 0.80-0.89).

Variable	Subject to Incorporation Bias?	Logistic Regression Coefficient	AUC when coefficient(s) set to zero	AUC 95% CI	Change in AUC
x1 SeptiScore	No	0.6375	0.75	0.70-0.89	-0.10
x2 PCT		0.0154	0.84	0.80-0.88	-0.01
x1 & x2 (jointly)			0.71	0.66-0.76	-0.14
x3 Temp (max)	Yes	0.6268	0.84	0.80-0.87	-0.01
x4 RR (max)		0.0366	0.85	0.81-0.88	-0.00
x5 MAP (max)		-0.0091	0.85	0.80-0.88	-0.00
x6 Age		0.0191	0.84	0.80-0.88	-0.01
x7 HR (min)		0.0073	0.85	0.81-0.88	-0.00

Of the variable subject to incorporation bias, exclusion of Temperature (max) produced the largest reduction in AUC (Δ AUC = −0.01), while exclusion of the remaining incorporation bias-sensitive variables resulted in smaller changes (|Δ AUC| < 0.01). These Δ AUC values represent upper bounds on the combined effect of each variable's legitimate predictive contribution and any inflation due to incorporation bias. Importantly, no single adjudication-incorporated variable (x3-x7) accounted for more than approximately 0.01 AUC units of the model's discrimination of sepsis vs. SIRS. This indicates that the observed AUC is not strongly driven by any individual variable suspected of incorporation bias and is relatively robust to their exclusion. While this analysis does not eliminate or quantify incorporation bias in absolute terms, it suggests that incorporation-related inflation is unlikely to be dominated by any single variable.

We additionally examined the joint effect of excluding all adjudication-incorporated variables by retaining only x1 (SeptiScore) and x2 (PCT) in the model. When the coefficients for x3–x7 were simultaneously set to zero, the resulting model yielded an AUC of 0.83 (95% CI 0.78-0.87), representing a reduction of 0.02 relative to the full seven-variable model.

This joint-exclusion analysis provides an upper bound on the combined contribution of variables subject to incorporation bias, encompassing both their legitimate predictive information and any inflation arising from their use in adjudication. Notably, the majority of the model's discriminative performance was preserved using only variables independent of the reference diagnosis, indicating that apparent discrimination is not primarily driven by adjudication-incorporated inputs.

Statistical conventions used in Tables S10 to S15.

Infection site independent model: For each cohort, time point and SeptiCyte RAPID inclusion/exclusion, one model is carried through Tables S10 to S15. It is the most parsimonious model attaining the maximum mean cross-validated AUC in the greedy search run without the suspected-site-of-infection indicators in the candidate pool. Infection sites having been excluded so that it could not confound variable selection. These models are therefore not selected in competition with the site-stratified searches of Figures 2 to 4 and Supplementary Figures S1 to S3, and no ranking against those searches is implied. As described in Section 2.5 of the manuscript, each cohort's infection site independent model was refit by logistic regression on its full mean-imputed sample (510k n = 419; Andalusian n = 353); all AUCs, confidence intervals and tests are DeLong [24], computed with pROC [22], and are apparent, in-sample values. Delta AUC is always the reduced model's AUC minus the full model's, so a negative value is discrimination lost. The nested-model caveat given in Section 2.5 applies to Tables S14, S14b and S14c, but not to Table S10, whose comparisons are non-nested. Reference numbers throughout this supplement refer to the reference list of the main manuscript.

The ten infection site independent models. These are the models whose performance is reported in Supplementary Tables S10 to S15.

Cohort	Time point	With or without SeptiCyte RAPID	Variables, in the order the greedy search selected them	No. of variables
510k	T1	Without	Temp (Max), RR (Max), MAP (Max), Age, HR (Min)	5
510k	T2	Without	PCT, SIRS criteria, RR (Max), SBP (qSOFA), Glucose (Max), Temp (Max), Age, HR (Min)	8
510k	T2	With	SeptiScore, SIRS criteria, RR (qSOFA), Age, SBP (qSOFA)	5
510k	T3	Without	PCT, Bacterial infection, Viral infection, BC culture, UC culture, SIRS criteria, SBP (qSOFA), RR (Max)	8
510k	T3	With	SeptiScore, Bacterial infection, SIRS criteria, Viral infection, BC culture, UC culture, SBP (qSOFA), Platelets (Min)	8
Andalusian	T1	Without	DBP, HR, Sex	3
Andalusian	T2	Without	PCT, CRP, DBP, Creatinine	4
Andalusian	T2	With	SeptiScore, DBP, Age, Creatinine	4
Andalusian	T3	Without	Bacterial infection, CRP, DBP, Fungal infection	4
Andalusian	T3	With	SeptiScore, Bacterial infection, DBP, Blood culture positive	4

Supplementary Table S10. DeLong tests comparing AUC across time points, both cohorts. Paired DeLong tests were estimated using the 'roc.test' function from the pROC R package [22] comparing AUC between successive time points and between SeptiCyte-excluded and SeptiCyte-

included models at T2 and T3, for the infection site independent model in each cohort refit on its full sample.

In every row, AUC A is the first model named in the Comparison column and AUC B the second; in the first row, for instance, AUC A is the T1 (−SeptiCyte RAPID) model and AUC B the T2 (+SeptiCyte RAPID) model. The Z is signed so that a negative value indicates that AUC B exceeds AUC A, and p is two-sided. The SeptiCyte-excluded and SeptiCyte-included models at each time point were selected by independent greedy searches and their variable sets differ substantially, so every comparison in this table is non-nested and the caveat given in the conventions note above does not apply to it.

**Cohort**	**Comparison**	**AUC A (first model)**	**AUC B (second model)**	**Z**	**p**
Andalusian	T1 vs. T2 (+SeptiCyte)	0.69	0.88	-5.247	1.5e-07
Andalusian	T2 (−SeptiCyte) vs. T2 (+SeptiCyte)	0.81	0.88	-2.573	0.010
Andalusian	T2 (+SeptiCyte) vs. T3 (+SeptiCyte)	0.88	0.93	-3.705	2.1e-04
Andalusian	T3 (−SeptiCyte) vs. T3 (+SeptiCyte)	0.90	0.93	-2.607	0.0091
510k	T1 vs. T2 (+SeptiCyte)	0.72	0.86	-5.064	4.1e-07
510k	T2 (−SeptiCyte) vs. T2 (+SeptiCyte)	0.79	0.86	-3.269	0.0011
510k	T2 (+SeptiCyte) vs. T3 (+SeptiCyte)	0.86	0.91	-3.772	1.6e-04
510k	T3 (−SeptiCyte) vs. T3 (+SeptiCyte)	0.87	0.91	-3.050	0.0023

Supplementary Table S11. Complete-case sensitivity analysis. All five models in both cohorts.

For the complete case sensitivity analysis, each infection site independent model per comparison was used to identify the patients with a complete set of observed (non-imputed) values for all variables. Because each model has a different predictor set, the retained subset and its 'n' differ. AUCs and their 95% confidence intervals are computed with pROC [22] (DeLong method). No variable selection was repeated: the formula is held fixed so that the comparison isolates the effect of imputation rather than confounding it with re-selection. Each model is estimated and evaluated as follows; the paired DeLong test compares columns 5 and 6, which are computed on identical patients.

(a) How each column is derived

Column #	Column Title	Model estimated from	AUC computed in
4	AUC (imputed model, full sample)	Full imputed sample	Full imputed sample
5	AUC (imputed model, complete cases)	Full imputed sample	Complete cases only
6	AUC (complete-case model)	Complete cases only	Complete cases only
7	Paired DeLong (cols 5 vs 6)	-	compares columns 5 and 6

(b) Results

Cohort	Model	Complete cases, n (%)	AUC (imputed model, full sample)	AUC (imputed model, complete cases)	AUC (complete-case model)	Paired DeLong (cols 5 vs 6)
Andalusian	T1	353/353 (100.0%)	0.69 (0.63-0.76)	0.69 (0.63-0.76)	0.69 (0.63-0.76)	Z = 0.000, p = 1.00
Andalusian	T2 (no SeptiCyte)	342/353 (96.9%)	0.81 (0.76-0.87)	0.82 (0.77-0.87)	0.82 (0.77-0.87)	Z = -0.642, p = 0.521
Andalusian	T2 (with SeptiCyte)	353/353 (100.0%)	0.88 (0.84-0.92)	0.88 (0.84-0.92)	0.88 (0.84-0.92)	Z = 0.000, p = 1.00
Andalusian	T3 (no SeptiCyte)	349/353 (98.9%)	0.90 (0.87-0.93)	0.90 (0.87-0.93)	0.90 (0.87-0.93)	Z = 0.000, p = 1.00
Andalusian	T3 (with SeptiCyte)	353/353 (100.0%)	0.93 (0.91-0.96)	0.93 (0.91-0.96)	0.93 (0.91-0.96)	Z = 0.000, p = 1.00
510k	T1	194/419 (46.3%)	0.72 (0.67-0.77)	0.69 (0.62-0.77)	0.70 (0.63-0.78)	Z = -0.564, p = 0.573
510k	T2 (no SeptiCyte)	130/419 (31.0%)	0.79 (0.74-0.83)	0.81 (0.73-0.89)	0.83 (0.76-0.90)	Z = -0.832, p = 0.405
510k	T2 (with SeptiCyte)	200/419 (47.7%)	0.86 (0.82-0.89)	0.88 (0.83-0.93)	0.89 (0.84-0.93)	Z = -1.365, p = 0.172
510k	T3 (no SeptiCyte)	183/419 (43.7%)	0.87 (0.84-0.91)	0.87 (0.82-0.92)	0.88 (0.83-0.93)	Z = -1.173, p = 0.241
510k	T3 (with SeptiCyte)	190/419 (45.3%)	0.91 (0.88-0.94)	0.91 (0.86-0.95)	0.91 (0.86-0.96)	Z = -1.535, p = 0.125

No comparison reaches significance (p = 0.125 to 1.00), giving no evidence that mean imputation altered discrimination in any reported model. Three Andalusian models retain 100% of patients, so for those models imputation is inert by construction.

Note on Table S11. For the 510k T2-no-SeptiCyte model, columns 4, 5 and 6 separate two effects that the headline figures conflate: restricting to the complete cases moves the AUC by +0.026 (column 5 − column 4), and re-estimating the model on them moves it by a further +0.015 (column 6 − column 5). No between-sample significance test is reported for the 419-vs-130 comparison itself: the complete cases are a strict subset of the full sample, so those two curves are neither paired nor independent and DeLong does not apply. As shown in Table S11b, complete-case restriction removes the VENUS and VENUS Supplement studies entirely, so the retained subset is drawn wholly from MARS (78 of 139 retained) and NEPTUNE (52 of 63 retained).

Supplementary Table S11b. Study composition of the 510k complete-case subset, illustrating that complete-case restriction removes the VENUS and VENUS Supplement studies entirely (representative 8-variable T2 model).

Complete cases are the patients with an observed, non-imputed value for every predictor in the 510k T2 (no SeptiCyte RAPID) infection site independent model, assessed in the raw pre-imputation data: 130 of 419 (31.0%). Study of origin was recovered by matching patient identifiers to the source data file. "% retained" is computed within study, "% of full cohort" is that study's share of all 419 patients, and "% of complete-case subset" its share of the 130. Each model in Table S11 defines a different subset; this one is shown because it is the most restrictive. VENUS and VENUS Supplement retain no patients here because several of the model's variables were not collected by protocol in those studies, so the loss is structural rather than random and the retained subset is drawn wholly from MARS and NEPTUNE.

Study	n total	n retained	% retained	% of full cohort	% of complete-case subset
MARS	139	78	56.1	33.2	60.0
NEPTUNE	63	52	82.5	15.0	40.0
VENUS	101	0	0.0	24.1	0.0
VENUS Supplement	116	0	0.0	27.7	0.0

Supplementary Table S11c. Differential-attenuation check: SeptiCyte RAPID increment re-estimated with no imputed values in either arm.

Method. SeptiScore has no missing values in either cohort, whereas several of its comparators do (Supplementary Tables S1 and S2). Because mean imputation attenuates a variable's association with the outcome, imputation could in principle favour SeptiScore mechanically in the with-versus-without comparison. Supplementary Table S11 cannot address this, since it evaluates each model on its own complete-case subset and never scores the two arms on the same patients. Here both models are restricted to the patients with fully observed values for every predictor in either model, refit on those patients, and compared by paired DeLong. No imputed value enters either arm, so neither model can be differentially attenuated.

Cohort	Comparison	Union complete cases, n (%)	AUC without SeptiCyte RAPID (95% CI)	AUC with SeptiCyte RAPID (95% CI)	Increment: imputed full sample → union complete cases	Paired DeLong
Andalusian	T2	342 / 353 (96.9%)	0.82 (0.77-0.87)	0.87 (0.83-0.92)	+0.066 → +0.055	Z = -2.161, p = 0.031
Andalusian	T3	349 / 353 (98.9%)	0.90 (0.87-0.93)	0.93 (0.91-0.96)	+0.033 → +0.031	Z = -2.488, p = 0.013

Cohort	Comparison	Union complete cases, n (%)	AUC without SeptiCyte RAPID (95% CI)	AUC with SeptiCyte RAPID (95% CI)	Increment: imputed full sample → union complete cases	Paired DeLong
510k	T2	130 / 419 (31.0%)	0.83 (0.76-0.90)	0.88 (0.82-0.94)	+0.070 → +0.051	Z = -1.659, p = 0.097
510k	T3	174 / 419 (41.5%)	0.87 (0.82-0.93)	0.91 (0.86-0.96)	+0.034 → +0.038	Z = -2.126, p = 0.033

Interpretation: The increment attributable to SeptiCyte RAPID persists in all four comparisons. Three shrink slightly, by 0.002 to 0.019 AUC units, and one (the 510k T3 model) increases by 0.003; the mean absolute change is about 0.009 and there is no consistent direction. Differential attenuation would predict a systematic reduction, largest in the 510k cohort where the missingness is concentrated, and that is not what is observed. Two cautions apply. The union subsets are smaller than the full samples, markedly so for 510k T2 (130 of 419), the confidence intervals widen and p-values rise even where the point estimate is essentially unchanged: the 510k T2 increment moves only from +0.0698 to +0.0512 while its p-value moves from 1.1×10^{-3} to 0.097. The Andalusian T2 comparison is significant in both views (p = 0.0101 imputed, p = 0.031 here), so the two agree. And the union subsets are not random samples of their cohorts (Supplementary Table S11b), so this is a test of the increment under equal treatment, not a re-estimate of absolute performance.

Supplementary Table S12. Variable-selection stability, 30 bootstrap resamples (Andalusian cohort, T2+SeptiCyte pool).

The greedy search was re-run independently on each of 30 bootstrap resamples of the Andalusian T2 (+SeptiCyte) variable pool.

Thirty resamples were drawn from the Andalusian cohort with replacement to the original sample size (n = 353), each with a fixed seed. Within each resample the greedy forward search was re-run from scratch to a maximum depth of six variables, selecting at each step the variable giving the highest apparent AUC. "Selected" is the percentage of resamples in which a variable entered at any step; "First pick" the percentage in which it entered first. Variables never selected are omitted. The candidate pool excludes the six suspected-site-of-infection indicators, which otherwise dominate the ranking. With 30 resamples the resolution of each percentage is 3.3 points, so the table indicates relative stability rather than precise frequencies.

Variable	Selected (% of 30 resamples)	First pick (%)
SeptiScore	100.0	93.3
DBP	83.3	0.0
age	63.3	0.0
pct	60.0	6.7
Creatinine	53.3	0.0
gender	30.0	0.0
All other variables	≤ 26.7	0.0

Supplementary Table S13. Variable-selection stability, 30 bootstrap resamples (510k cohort, T2+SeptiCyte pool).

The greedy search was re-run independently on each of 30 bootstrap resamples of the 510k T2 (+SeptiCyte) variable pool. As Table S12, applied to the 510k cohort's T2 (+SeptiCyte RAPID) pool (n = 419), which likewise excludes the site-of-infection indicators.The search depth here is seven variables

rather than the six used for Table S12, matching each cohort's own infection site independent model size. The cautions given for Table S12 apply unchanged.

Variable	Selected (% of 30 resamples)	First pick (%)
SeptiScore	100.0	100.0
SIRS.criteria	73.3	0.0
Age	46.7	0.0
RR_qSOFA	46.7	0.0
Respiratory.Rate.Min	43.3	0.0
Mean.Art.Pressure.Max	40.0	0.0
PCT	36.7	0.0
All other variables	≤ 30.0	0.0

Supplementary Table S14. Incorporation-bias variable-exclusion analysis extended to the Andalusian T2/T3 and 510k T1/T3 models.

Extension of the Section 3.8 / Table S9 sensitivity analysis to additional models, quantifying the AUC contribution of variable subsets subject to incorporation bias. For each row the infection site independent model was refit on the named subset of its variables, using the same patients and the same mean-imputed data, and the apparent AUC recomputed; p is a paired DeLong test against the full model. Subsets are specified in advance rather than re-searched, so the comparison is not confounded with re-selection. These are nested comparisons; see the conventions note preceding Table S10. The 510k T2 model is not repeated here; the equivalent analysis for that time point is given in Section 3.8, Supplementary Figure S4 and Supplementary Table S9, which use a seven-variable greedy model (SeptiCyte RAPID, PCT, Temp (max), RR (max), MAP (max), age, HR (min); AUC 0.85) rather than the infection site independent model carried through Tables S10 to S15.

Cohort	Model	Variable set	AUC	Delta vs. full	p (vs. full)
Andalusian	T2	full (4 vars)	0.88	-	-
Andalusian	T2	SeptiScore only	0.84	-0.039	0.0090
Andalusian	T2	clinical vars only (no SeptiScore)	0.67	-0.211	5.9e-09
Andalusian	T3	full (4 vars)	0.93	-	-
Andalusian	T3	SeptiScore only	0.84	-0.094	1.0e-05
Andalusian	T3	microbiology vars only	0.81	-0.126	1.8e-15
510k	T3	full (8 vars)	0.91	-	-
510k	T3	SeptiScore only	0.82	-0.084	3.7e-07

Supplementary Table S14b. 510k T3 leave-one-out detail (full model AUC = 0.91).

Method for Tables S14b and S14c. Each variable in turn was dropped from the full model, the model refit on the same patients and the same mean-imputed data, and the apparent AUC recomputed; p is a paired DeLong test against the full model. Rows are ordered by decreasing loss of AUC.

Variable dropped from full model	AUC after drop	Delta vs. full	p (vs. full)
SeptiScore	0.86	-0.051	9.7e-05
Bacterial	0.90	-0.010	0.097
Viral	0.90	-0.010	0.075
SIRS.criteria	0.90	-0.006	0.285
BC_culture	0.90	-0.006	0.105
UC_culture	0.90	-0.005	0.149
SBP_qSOFA	0.90	-0.003	0.356
Platelets.Min	0.90	-0.002	0.547

Supplementary Table S14c. 510k T1 leave-one-out detail (full model AUC = 0.72).

As Table S14b. The T1 candidate pool contains no SeptiCyte RAPID, site-of-infection or qSOFA variable, so this leave-one-out covers vital signs and demographics only.

Variable dropped from full model	AUC after drop	Delta vs. full	p (vs. full)
Temp.Max	0.66	-0.052	0.012
Age	0.70	-0.020	0.122
HeartRate.Min	0.70	-0.019	0.090
Respiratory.Rate.Max	0.70	-0.017	0.127
Mean.Art.Pressure.Max	0.71	-0.011	0.277

Supplementary Table S15. Sensitivity and specificity of the infection site independent models at their Youden-optimal cutoffs, both cohorts.

Sensitivity and specificity of each infection site independent model at its Youden-optimal cutoff, the threshold maximising sensitivity + specificity − 1. Likelihood ratios and post-test probabilities are not reported for these cohorts. A likelihood ratio is a ratio whose denominator is 1 − specificity, so its sampling error is amplified relative to that of sensitivity and specificity, severely so as specificity approaches 1, and the interval is unbounded when no false positives are observed; attaining a given precision for a likelihood ratio therefore requires a substantially larger sample than for the underlying proportions [45]. Likelihood ratios for SeptiScore are instead referred to from [36], a combined cohort of 889 critically ill patients recruited across 19 hospitals in the USA and Europe, rather than the 419 (510k) and 353 (Andalusian) patients analysed here.

Cohort	Model	Se	Sp
Andalusian	T1 (−SeptiCyte)	0.61	0.73
Andalusian	T2 (+SeptiCyte)	0.85	0.83
Andalusian	T3 (+SeptiCyte)	0.94	0.77
510k	T1 (−SeptiCyte)	0.64	0.71
510k	T2 (+SeptiCyte)	0.74	0.83

Cohort	Model	Se	Sp
510k	T3 (+SeptiCyte)	0.78	0.90
